# Blood–brain barrier dysfunction and peripheral immune activation in Alzheimer’s disease: an inflammation-centered review

**DOI:** 10.3389/fimmu.2026.1955233

**Published:** 2026-09-09

**Authors:** Zuli Yang, Zhiqiang Xiao, Bin Huang, Xiaoping Wang

**Affiliations:** 1 Department of Neurology, Chengdu University of Traditional Chinese Medicine, Chengdu, China; 2 Department of Neurology, Sichuan Provincial People’s Hospital, University of Electronic Science and Technology of China, Chengdu, China

**Keywords:** Alzheimer’s disease, blood–brain barrier, monocytes, neuroinflammation, peripheral immunity

## Abstract

Alzheimer’s disease (AD) is traditionally defined by amyloid-β deposition, tau pathology, synaptic failure, and progressive cognitive decline. However, growing evidence indicates that neurovascular dysfunction and systemic immune activation are not merely secondary consequences but active contributors to disease progression. The blood–brain barrier (BBB), as a dynamic immunovascular interface, regulates the communication between the central nervous system and the peripheral immune system. In AD, aging, amyloid-β toxicity, tau-related stress, vascular senescence, endothelial inflammatory signaling, pericyte injury, and gliovascular remodeling can weaken BBB integrity. Barrier disruption may then permit peripheral cytokines, chemokines, plasma-derived factors, monocytes, and T-cell–related signals to influence microglial activation, astrocyte reactivity, oxidative stress, synaptic dysfunction, and neuronal injury. Recent studies further suggest that peripheral monocytes may participate in amyloid-β clearance and transport, whereas chronic monocyte activation, adaptive immune remodeling, and T-cell exhaustion may amplify neuroinflammation. This review summarizes recent experimental and clinical evidence linking BBB dysfunction, peripheral immune activation, and AD progression. We propose an inflammation-centered model in which BBB breakdown and peripheral immune dysregulation form a pathogenic loop that accelerates neurodegeneration. Targeting this BBB–peripheral immune axis may provide new opportunities for biomarker development, patient stratification, and combination therapy beyond classical amyloid- and tau-directed strategies.

## Highlights

- BBB dysfunction links vascular injury, systemic inflammation, and AD progression.
- Peripheral monocytes and T cells may shape neuroinflammation in AD.
- Integrated BBB–immune biomarkers may support vascular-inflammatory phenotyping in AD.

## 1 Introduction

Alzheimer’s disease (AD) is the most common cause of dementia and is pathologically characterized by extracellular amyloid-β (Aβ) deposition, intracellular tau aggregation, synaptic dysfunction, neuronal loss, and progressive cognitive decline (1–4). For decades, the amyloid cascade and tau propagation hypotheses have dominated mechanistic interpretations of AD. However, increasing clinical, pathological, imaging, and experimental evidence indicates that AD cannot be fully explained by neuronal proteinopathy alone. Instead, AD should be viewed as a multifactorial disorder in which protein aggregation, neuroinflammation, vascular dysfunction, metabolic stress, glial activation, and immune dysregulation interact across disease stages (5–9).

Among these non-neuronal mechanisms, cerebrovascular dysfunction and blood–brain barrier (BBB) breakdown have gained increasing attention. The BBB is a highly specialized vascular interface that maintains central nervous system (CNS) homeostasis by regulating molecular transport, preventing uncontrolled entry of blood-derived factors, supporting immune surveillance, and facilitating waste clearance (10–14). In AD, disruption of this barrier may expose the brain parenchyma to plasma proteins, inflammatory cytokines, chemokines, leukocyte-derived mediators, and other peripheral signals that can amplify neuroinflammation and neuronal injury. Importantly, BBB dysfunction may not simply represent a late consequence of neurodegeneration. Rather, it may actively participate in disease initiation and progression by impairing Aβ clearance, promoting vascular inflammation, disturbing neurovascular coupling, and enhancing glial reactivity.

Recent studies have provided stronger evidence linking BBB impairment with cognitive dysfunction and AD-related pathological changes. Clinical evidence has connected BBB abnormalities with human cognitive impairment and AD, supporting the concept that vascular barrier damage is clinically relevant rather than merely incidental (15). Moreover, BBB permeability has been associated with distinct neuroinflammatory profiles in AD, suggesting that barrier dysfunction may help define inflammatory heterogeneity among patients (16). More recently, BBB-related biomarkers were shown to modulate the associations between peripheral immune markers and AD pathology, brain atrophy, and cognitive performance, further strengthening the idea that BBB dysfunction acts as a biological bridge between systemic inflammation and neurodegeneration (17). Together, these findings support an inflammation-centered vascular perspective in which BBB breakdown and immune activation are integral components of AD pathophysiology.

The BBB has traditionally been described as a physical and biochemical barrier that protects the brain from potentially harmful circulating substances. However, this view is incomplete. The BBB is also an immunologically active interface that continuously communicates with peripheral immune cells, cytokines, chemokines, complement components, and metabolic signals. Brain endothelial cells express adhesion molecules, transporters, receptors, and inflammatory signaling pathways that allow them to respond dynamically to systemic immune changes. Under physiological conditions, this regulated interface preserves CNS immune privilege while permitting controlled immune surveillance. Under pathological conditions, however, BBB dysfunction can transform the neurovascular unit into a site of inflammatory amplification. In AD, BBB disruption may allow peripheral immune mediators to influence the CNS through several non-mutually exclusive mechanisms (18–22). First, increased BBB permeability may permit the entry of plasma-derived proteins, inflammatory cytokines, chemokines, and damage-associated molecular signals into the perivascular and parenchymal compartments. These signals can activate microglia and astrocytes, induce oxidative stress, and impair synaptic function. Second, endothelial activation can increase the expression of adhesion molecules and chemotactic factors, thereby facilitating leukocyte adhesion, perivascular accumulation, or limited parenchymal infiltration. Third, impaired BBB transport systems may reduce Aβ efflux and disturb metabolic exchange, further promoting protein accumulation and inflammatory stress. Fourth, BBB-related changes may alter communication between the CNS and peripheral immune system even in the absence of massive leukocyte infiltration, because endothelial and perivascular cells can transmit inflammatory signals across the barrier.

Peripheral innate immunity is especially relevant to this axis. Peripheral monocytes may participate in Aβ binding, clearance, and transport, and their functional status appears to correlate with AD progression (23). In mild cognitive impairment (MCI), large Aβ aggregates in plasma can activate peripheral monocytes, suggesting that peripheral immune activation may begin during prodromal disease stages rather than only after dementia has developed (24). These observations indicate that the periphery is not a passive compartment in AD. Instead, circulating immune cells may sense AD-related protein aggregates, respond to systemic inflammatory cues, and communicate with the CNS through vascular and barrier-dependent mechanisms (25–29). Adaptive immunity is also increasingly recognized as part of AD progression. T-cell exhaustion has been associated with cognitive status and amyloid accumulation, implying that chronic immune stimulation or immune aging may accompany AD pathology (30). In addition, broader adaptive immune changes have been linked to clinical progression of AD, including alterations in peripheral T-cell and B-cell compartments (31). These findings raise the possibility that AD involves a systemic immune remodeling process, in which peripheral immune changes interact with BBB dysfunction and CNS inflammation. Therefore, focusing on the BBB–peripheral immune axis provides a useful framework for integrating vascular pathology, systemic inflammation, glial activation, and neurodegeneration.

This review examines the inflammation-centered interplay among BBB dysfunction, neurovascular unit injury, peripheral immune activation, and AD progression. Rather than considering vascular and systemic immune abnormalities as separate processes, we propose that AD-related proteinopathy, aging, vascular stress, endothelial inflammation, and immune remodeling interact through the BBB–neurovascular interface to form a self-reinforcing pathogenic axis. We summarize clinical, experimental, and multi-omics evidence linking BBB disruption with cognitive impairment, neuroinflammatory heterogeneity, and AD pathology, and discuss key mechanisms including Aβ-induced pericyte injury, Wnt/β-catenin dysregulation, type I interferon signaling, vascular senescence, DR6, and ANGPT2. We further examine peripheral monocyte-mediated Aβ handling, myeloid-cell recruitment, T-cell dysfunction, and adaptive immune remodeling across disease stages, with particular emphasis on how BBB status may modify the impact of peripheral immunity on the CNS. Integrating evidence from human cohorts, animal models, cellular systems, and multi-omics studies, we propose a testable vascular-inflammatory AD phenotype characterized by concurrent neurovascular dysfunction and peripheral immune dysregulation. This framework may support biomarker development, patient stratification, and therapeutic strategies complementary to conventional Aβ- and tau-directed approaches.

## 2 The BBB and neurovascular unit in Alzheimer’s disease

### 2.1 Structure and function of the BBB in the healthy brain

The BBB is a specialized vascular barrier formed primarily by brain microvascular endothelial cells, which are connected by tight junctions and adherens junctions (32, 33). These endothelial cells differ from peripheral endothelial cells by having low rates of transcytosis, high electrical resistance, polarized transporter expression, and specialized metabolic and immune-regulatory functions. Tight junction proteins restrict paracellular diffusion, while transporters and receptors regulate the selective movement of nutrients, metabolites, peptides, and waste products between the blood and the CNS. However, the BBB does not function in isolation. It is embedded within the neurovascular unit, which includes endothelial cells, pericytes, astrocytic endfeet, basement membrane, vascular smooth muscle cells, microglia, neurons, and perivascular immune components. Pericytes regulate vascular stability, capillary diameter, endothelial gene expression, and BBB integrity. Astrocytic endfeet ensheath cerebral microvessels and contribute to water transport, metabolic support, and vascular signaling. Microglia and perivascular macrophages participate in immune surveillance and inflammatory responses. Together, these cell types maintain CNS homeostasis, regulate neurovascular coupling, and coordinate responses to metabolic or inflammatory stress.

In the context of AD, this multicellular organization is particularly important because Aβ accumulation, tau pathology, hypoperfusion, oxidative stress, and inflammation may affect multiple components of the neurovascular unit simultaneously. Recent single-nucleus transcriptomic analyses have identified vascular-cell alterations associated with impaired angiogenesis and BBB function in AD, highlighting endothelial and vascular-associated cell states that may contribute to disease-related barrier dysfunction (34). In parallel, gliovascular transcriptomic studies have revealed altered interactions among vascular and glial cell populations, including disrupted endothelial–pericyte–astrocyte communication and molecular changes related to BBB dysfunction (35). These findings support the view that BBB impairment in AD is not merely the result of endothelial damage alone, but rather reflects broader neurovascular unit remodeling. The BBB also contributes to Aβ homeostasis. Under physiological conditions, Aβ can be cleared from the brain through multiple pathways, including receptor-mediated transport across the BBB, perivascular drainage, glymphatic clearance, and cellular uptake by resident or peripheral phagocytes. When BBB transport systems and vascular integrity are compromised, Aβ clearance may decline, while vascular inflammation and barrier leakage increase. This creates a self-reinforcing cycle in which Aβ promotes vascular injury, BBB dysfunction reduces Aβ clearance, and persistent neurovascular stress accelerates neuroinflammation.

### 2.2 Clinical evidence of BBB disruption in cognitive impairment and AD

Clinical studies increasingly support the association between BBB dysfunction and cognitive impairment. BBB disruption has been linked with human cognitive impairment and AD, suggesting that cerebrovascular barrier damage may be relevant to clinical manifestations of neurodegeneration (15). Such evidence is important because it shifts BBB dysfunction from a purely experimental concept to a clinically measurable feature of AD-related decline. In patients, BBB damage may be reflected by imaging-based permeability measures, cerebrospinal fluid or blood biomarkers of endothelial injury, albumin ratio changes, inflammatory mediator profiles, and neurovascular markers (36–39). BBB permeability may also help explain the heterogeneity of neuroinflammation in AD. One study reported that BBB permeability is associated with different neuroinflammatory profiles in AD, suggesting that patients with greater barrier dysfunction may display distinct inflammatory signatures (16). This finding is highly relevant for patient stratification. AD is clinically and biologically heterogeneous; some patients may show predominant amyloid/tau pathology, while others may have stronger vascular, inflammatory, metabolic, or immune components. BBB permeability may therefore function as a marker of a vascular-inflammatory AD subtype.

The relationship between BBB dysfunction and peripheral immunity has also been supported by biomarker-based clinical evidence. A recent study showed that BBB-related biomarkers modulate associations between peripheral immune markers and AD-related outcomes, including AD pathology, cerebral atrophy, and cognitive function (17). In that framework, peripheral immune alterations are not considered isolated systemic findings. Instead, their influence on the brain may depend partly on BBB status. This is conceptually important because it suggests that BBB integrity may determine how strongly peripheral inflammation is translated into CNS injury. Clinically, BBB dysfunction may contribute to cognitive impairment through several mechanisms. Leakage of plasma proteins into the brain can activate glial cells and disturb synaptic homeostasis. Endothelial inflammation can impair vasodilatory responses and reduce neurovascular coupling, leading to metabolic mismatch during neuronal activity. Pericyte dysfunction can destabilize capillaries and worsen hypoperfusion. Altered transporter expression can reduce clearance of Aβ and other toxic metabolites. Together, these processes can promote synaptic injury, neuronal vulnerability, and cognitive decline. Therefore, BBB disruption should be considered not only a vascular biomarker but also a mechanistic contributor to AD progression.

### 2.3 BBB dysfunction as an early and dynamic event

A key unresolved question is whether BBB dysfunction precedes amyloid and tau pathology, develops in parallel with them, or emerges as a downstream consequence of neurodegeneration. Available evidence does not support a single temporal sequence applicable to all patients. Instead, BBB dysfunction may arise through partially distinct trajectories depending on age, APOE genotype, vascular burden, inflammatory status, and the stage of AD pathology. Importantly, human imaging studies suggest that BBB breakdown can be detected during early cognitive dysfunction and may occur independently of established Aβ and tau abnormalities. Nation et al. reported increased hippocampal BBB permeability in individuals with early cognitive impairment independent of classical Aβ and tau biomarkers (40). Similarly, APOE4-associated BBB breakdown in the hippocampus and parahippocampal gyrus predicted cognitive decline independently of CSF Aβ and tau status, supporting the possibility that vascular-barrier dysfunction can precede or develop independently of overt proteinopathy in a subset of individuals (41).

Other evidence supports a parallel or bidirectional relationship between BBB dysfunction and Aβ/tau pathology. In patients with cognitive impairment, BBB permeability has been associated with AD-related biomarkers, although these relationships vary according to amyloid status. For example, cortical BBB permeability showed differential associations with CSF tau, neuronal injury, and Aβ-related measures in amyloid-positive and amyloid-negative individuals (42). More recent longitudinal observations in cognitively normal older adults further showed that greater hippocampal BBB disruption was associated with worse episodic memory independently of Aβ and tau burden, whereas faster preceding entorhinal tau accumulation was also associated with greater subsequent BBB disruption (43). These findings suggest that BBB dysfunction and proteinopathy may evolve in parallel and interact dynamically rather than following a strictly unidirectional sequence. Aging is one of the strongest risk factors for AD and is closely associated with vascular dysfunction, endothelial stress, and chronic low-grade inflammation. Vascular senescence may provide a mechanistic bridge between aging and BBB breakdown. Experimental evidence indicates that vascular senescence and leak are early features of BBB breakdown in AD models, with senescent vascular cells contributing to barrier instability (44). This supports the idea that aging-related endothelial and pericyte dysfunction may prime the BBB before extensive neurodegeneration is established.

Inflammatory endothelial activation may further accelerate BBB impairment. In AD, Aβ, tau-related stress, oxidative injury, systemic inflammation, and vascular risk factors may induce endothelial inflammatory programs that weaken tight junctions, alter transporter activity, and increase vascular permeability (45–48). Recent work identified angiopoietin-2 as a mediator that aggravates AD by promoting BBB dysfunction and neuroinflammation, further supporting the role of endothelial signaling in disease progression (49). Such findings suggest that BBB dysfunction is not a passive leak phenomenon but an active molecular process involving vascular inflammatory mediators. BBB dysfunction may also interact bidirectionally with neuroinflammation. Increased BBB permeability may allow peripheral inflammatory signals to enter or influence the CNS, thereby activating microglia and astrocytes. In turn, activated glial cells can release cytokines, chemokines, reactive oxygen species, and matrix-remodeling enzymes that further damage the neurovascular unit. This reciprocal process may create a pathogenic loop: vascular injury promotes inflammation, and inflammation worsens vascular injury. Clinical evidence linking BBB permeability with different neuroinflammatory profiles in AD supports this bidirectional model (16).

**Figure 1:**
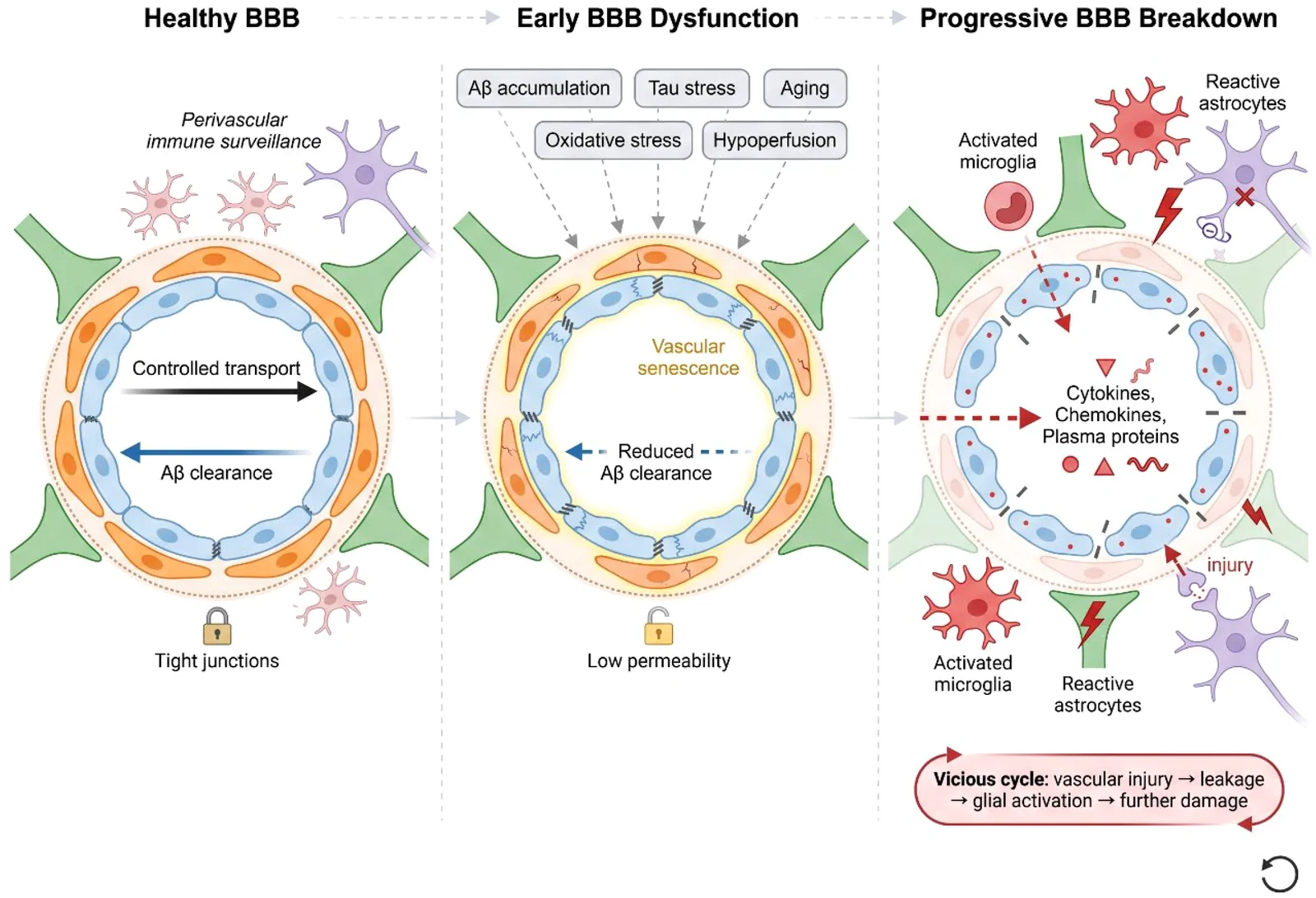
BBB dysfunction and peripheral immune activation in Alzheimer’s disease. A healthy BBB/neurovascular unit maintains CNS homeostasis and supports Aβ clearance. In AD, Aβ accumulation, tau-related stress, aging, oxidative stress, and inflammation injure endothelial cells and pericytes, disrupt tight junctions, and reduce Aβ clearance. Progressive BBB leakage permits peripheral inflammatory mediators and immune signals to amplify glial activation, synaptic dysfunction, neuronal injury, and cognitive decline.

The role of peripheral immunity further strengthens the concept of BBB dysfunction as a dynamic event. If BBB biomarkers modulate the association between peripheral immune markers and AD pathology or cognition, then BBB status may influence when and how systemic inflammation contributes to brain injury (17). In early disease, subtle BBB dysfunction may be sufficient to increase inflammatory signaling across the vascular interface without obvious leukocyte infiltration. As disease progresses, more severe barrier disruption may permit greater plasma protein leakage, stronger endothelial activation, and enhanced immune-cell communication with the CNS. Taken together, current evidence does not support a single fixed temporal position for BBB dysfunction within the AD cascade. In some individuals, particularly those with vascular vulnerability or APOE4-associated pericyte injury, BBB disruption may precede detectable or advanced Aβ/tau pathology. In others, BBB dysfunction may develop concurrently with amyloid deposition, tau accumulation, and neuroinflammation, with each process reinforcing the others. At later stages, established Aβ/tau pathology, glial activation, oxidative stress, and vascular inflammation may further aggravate BBB breakdown. BBB dysfunction should therefore be viewed as a stage- and context-dependent process that can act as an early susceptibility factor, a parallel pathological component, and a downstream amplifier of AD progression. **Figure 1** illustrates the progression from a healthy BBB/neurovascular unit to vascular-inflammatory barrier dysfunction in AD. It highlights how BBB leakage may connect peripheral immune activation with neuroinflammation, synaptic injury, and cognitive decline.

## 3 Molecular mechanisms driving BBB dysfunction in AD

### 3.1 Aβ-induced pericyte injury and BBB breakdown

Aβ deposition is a central pathological feature of AD, but its pathogenic effects are not restricted to neurons or plaques within the brain parenchyma. Aβ can also directly impair the neurovascular unit, particularly pericytes, which are essential regulators of BBB integrity, capillary stability, vascular tone, and endothelial function (50–54). Pericytes are embedded within the vascular basement membrane and closely interact with brain endothelial cells and astrocytic endfeet. Through these interactions, they help maintain tight junction organization, suppress endothelial transcytosis, regulate capillary blood flow, and support the structural stability of cerebral microvessels. Therefore, pericyte injury can weaken the BBB even before widespread vascular collapse occurs. Recent experimental evidence has linked Aβ-induced pericyte injury to mitochondrial dysfunction and ferroptosis. Aβ1–40 was shown to induce BBB destruction by promoting mitophagy-dependent ferroptosis in pericytes through the CD36/PINK1/Parkin pathway (55). This mechanism is important because it connects vascular Aβ toxicity with two interrelated forms of cellular stress: mitochondrial quality-control disruption and iron-dependent lipid peroxidation. CD36, a scavenger receptor involved in vascular Aβ responses, can facilitate Aβ-related oxidative and inflammatory injury. Activation of the PINK1/Parkin pathway indicates that damaged mitochondria are being targeted for mitophagy; however, excessive or dysregulated mitophagy may contribute to mitochondrial depletion, bioenergetic failure, and cellular vulnerability. When this process is coupled with ferroptosis, pericytes become susceptible to lipid peroxidation, iron dyshomeostasis, and oxidative membrane damage.

The consequence of pericyte ferroptotic injury is BBB destabilization. Loss or dysfunction of pericytes can reduce endothelial support, disrupt tight junction integrity, increase vascular permeability, and impair capillary regulation (56–60). In AD, this may create a self-amplifying loop. Aβ accumulation injures pericytes and weakens the BBB; BBB breakdown then impairs Aβ clearance and exposes the brain to blood-derived inflammatory mediators; these events further activate glial cells and exacerbate neurovascular damage. Thus, pericytes represent a key cellular bridge between amyloid pathology and vascular dysfunction. The CD36/PINK1/Parkin–mitophagy–ferroptosis axis also provides a mechanistic framework for understanding why vascular Aβ deposition and BBB breakdown may occur together in AD. This pericyte-centered mechanism expands the classical amyloid hypothesis by emphasizing that Aβ can act as a vascular toxin. Instead of viewing Aβ only as a trigger of synaptic dysfunction and neuronal death, it is necessary to consider its direct effects on the cells that maintain vascular and barrier homeostasis. Pericyte injury may be particularly relevant in early or vascular-inflammatory forms of AD, where BBB leakage, cerebral hypoperfusion, and neuroinflammation coexist with amyloid pathology.

### 3.2 Endothelial Wnt/β-catenin signaling as a BBB-protective pathway

Wnt/β-catenin signaling is a critical pathway for BBB development, maturation, and maintenance. In brain endothelial cells, canonical Wnt signaling promotes BBB-specific gene expression, tight junction organization, transporter regulation, and suppression of vascular leakage (61, 62). The pathway is initiated by Wnt ligands binding to Frizzled receptors and co-receptors such as LRP5/6, leading to β-catenin stabilization and transcriptional activation of BBB-supportive genes. When this pathway is impaired, endothelial cells may lose their specialized barrier phenotype and become more permeable or inflammatory (63–66). In AD, Aβ can suppress endothelial Wnt/β-catenin signaling and thereby contribute to BBB malfunction. Activation of the Wnt/β-catenin pathway was shown to mitigate BBB dysfunction in AD, and targeting LRP6 in brain endothelial cells alleviated Aβ-induced BBB impairment (67). This study is particularly important because it identifies an endothelial signaling mechanism that is not merely associated with BBB dysfunction but can be experimentally manipulated to restore barrier properties. By activating LRP6-dependent Wnt/β-catenin signaling, endothelial cells may regain expression of BBB functional proteins and improve barrier integrity in the presence of Aβ stress. This mechanism suggests that BBB dysfunction in AD is at least partly reversible at the signaling level. Aβ-induced endothelial injury does not necessarily represent irreversible vascular degeneration; instead, it may reflect suppression of protective endothelial programs. Restoring Wnt/β-catenin signaling could stabilize tight junctions, reduce paracellular leakage, improve endothelial identity, and potentially support Aβ clearance across the vascular interface. Therefore, Wnt/β-catenin signaling represents a vascular-protective pathway with therapeutic relevance.

The Wnt/β-catenin pathway may also interact with other mechanisms discussed in this review. For example, endothelial inflammatory signaling, oxidative stress, senescence, and Aβ exposure may converge to reduce BBB-protective transcriptional programs (68, 69). Conversely, activation of Wnt/β-catenin signaling may counteract some aspects of endothelial dysfunction, including barrier leakiness and loss of tight junction proteins (70). This makes the pathway a central node in the balance between BBB injury and repair. From a translational perspective, selective modulation of Wnt signaling in brain endothelial cells may be more feasible than global pathway activation, because Wnt signaling has broad effects on vascular development, angiogenesis, cell proliferation, and tissue homeostasis (71). Future studies should clarify whether endothelial-specific Wnt activation can safely preserve BBB function in AD without causing unwanted angiogenic or proliferative effects.

### 3.3 Type I interferon signaling and inflammatory endothelial dysfunction

Inflammatory cytokine signaling is another major driver of BBB dysfunction in AD. Among inflammatory pathways, type I interferon signaling has emerged as an important contributor to brain endothelial barrier impairment. Type I interferons, including IFN-α and IFN-β, are classically involved in antiviral defense, but persistent activation of this pathway can induce chronic inflammatory states, alter endothelial function, and amplify immune responses. In the aging or AD brain, type I interferon signaling may be triggered by nucleic acid sensing, glial activation, cellular stress, Aβ-associated inflammation, or systemic immune activation (72–76). Experimental evidence indicates that increased type I interferon signaling contributes to brain endothelial barrier dysfunction in an AD model (77). In human brain endothelial cells, IFN-β stimulation reduced the levels of VE-cadherin, occludin, and claudin-5, which are essential components of adherens and tight junction structures. The reduction of these junctional proteins was accompanied by increased endothelial leakiness. Importantly, depletion of the type I interferon receptor prevented IFN-β-induced VE-cadherin downregulation and restored endothelial barrier integrity (78–80). These findings provide a direct mechanistic link between inflammatory cytokine signaling and structural disruption of the endothelial barrier. The relevance of this pathway lies in its ability to connect systemic or CNS inflammation with BBB leakage. Type I interferon signaling may convert brain endothelial cells from a barrier-maintaining phenotype into a more inflammatory and permeable state. Loss of VE-cadherin can destabilize endothelial cell–cell adhesion, while reduced occludin and claudin-5 weaken tight junction integrity. As a result, blood-derived factors may more easily enter the perivascular space or brain parenchyma. This may amplify microglial and astrocytic activation, increase oxidative stress, and promote neuronal vulnerability.

Type I interferon signaling may also enhance immune communication across the BBB (81–86). Activated endothelial cells can upregulate chemokines, adhesion molecules, and inflammatory mediators, thereby facilitating leukocyte-endothelial interactions and immune-cell recruitment. Even without large-scale immune-cell infiltration, endothelial inflammatory activation can transmit peripheral inflammatory signals to the CNS. In AD, this is especially relevant because chronic low-grade inflammation, aging-related immune dysregulation, and Aβ-induced glial activation may sustain interferon-associated vascular stress. Thus, type I interferon signaling provides a mechanistic example of how inflammation can directly damage the BBB and convert the neurovascular interface into an amplifier of AD pathology.

### 3.4 Vascular senescence and age-related BBB leakage

Aging is the strongest risk factor for AD, and vascular aging may be one of the major biological processes linking age to neurodegeneration (87, 88). With advancing age, endothelial cells, pericytes, vascular smooth muscle cells, and perivascular immune cells undergo structural and functional changes (89, 90). These include impaired mitochondrial function, oxidative stress, reduced nitric oxide bioavailability, basement membrane thickening, reduced vascular plasticity, altered transporter expression, and increased inflammatory signaling (89, 91, 92). Collectively, these changes weaken the neurovascular unit and may lower the threshold for BBB breakdown in response to Aβ, tau, or systemic inflammation (87, 93). Cellular senescence is a particularly important feature of vascular aging. Senescent cells permanently exit the cell cycle but remain metabolically active and often secrete inflammatory cytokines, chemokines, matrix-remodeling enzymes, growth factors, and reactive oxygen species (94–99). This secretory program, often termed the senescence-associated secretory phenotype, can propagate inflammation and tissue dysfunction. In the cerebral vasculature, senescent endothelial cells or perivascular cells may disrupt barrier integrity, promote vascular leak, impair angiogenic responses, and activate neighboring glial cells.

Evidence from AD patients and mouse models indicates that vascular senescence and leak are features of early BBB breakdown (44). This finding is conceptually important because it suggests that BBB impairment can emerge as part of an aging-related vascular process rather than only as a late consequence of neuronal degeneration. Senescent vascular cells may prime the BBB for dysfunction before extensive plaque burden or neuronal loss becomes dominant (100–104). Once vascular leak begins, plasma-derived proteins and inflammatory mediators can enter the brain environment, further promoting glial activation and neuroinflammatory damage. The concept of vascular senescence also connects BBB dysfunction with systemic inflammaging. Aging is associated with chronic low-grade inflammation in the periphery, including increased circulating cytokines, immune-cell dysfunction, and altered myeloid and lymphoid responses. If the BBB is already weakened by vascular senescence, systemic inflammatory signals may have a greater impact on the CNS. This may partly explain why older individuals with vascular risk factors, chronic inflammatory conditions, or immune dysregulation are more vulnerable to cognitive decline. In this model, vascular senescence acts as a permissive state that allows Aβ/tau pathology and peripheral inflammation to more effectively damage the brain.

Targeting senescence-related vascular dysfunction may therefore represent an attractive preventive or early-intervention strategy. Potential approaches include reducing oxidative stress, improving endothelial metabolism, modulating senescence-associated inflammatory programs, preserving pericyte function, and restoring vascular repair mechanisms. However, future work is needed to determine whether vascular senescence is a cause, consequence, or parallel feature of AD progression. Longitudinal human studies combining BBB imaging, vascular biomarkers, inflammatory profiling, and amyloid/tau biomarkers will be essential to define the temporal sequence.

### 3.5 DR6 and ANGPT2 as emerging endothelial regulators of BBB integrity

Recent studies have identified additional endothelial regulators that may contribute to BBB dysfunction in AD. Among them, death receptor 6 (DR6) and angiopoietin-2 (ANGPT2) are particularly relevant because they connect endothelial stress, vascular signaling, and neuroinflammation. DR6 is a member of the tumor necrosis factor receptor superfamily and has been implicated in neuronal and vascular biology. In AD-related BBB malfunction, endothelial DR6 appears to play a regulatory role in maintaining barrier integrity (105). Experimental evidence showed that brain endothelial DR6 levels were altered in AD models and that Aβ exposure affected DR6 expression in cultured brain endothelial cells (106). Suppression of DR6 worsened BBB malfunction in the presence of Aβ, whereas DR6 overexpression improved BBB functional protein expression and rescued barrier dysfunction *in vitro*. Mechanistically, DR6 may interact with Wnt/β-catenin and JNK-related pathways, suggesting that it is not simply a death receptor but may also participate in endothelial adaptive responses. This finding is important because it identifies endothelial DR6 as a potential BBB-protective factor in early AD-related vascular stress. If DR6 supports BBB functional protein expression, then reduced DR6 signaling may make endothelial cells more vulnerable to Aβ-induced dysfunction. This also suggests that AD-related endothelial injury may involve the loss of protective signaling mechanisms, rather than only activation of damaging pathways. DR6 may therefore represent a candidate target for restoring endothelial resilience.

ANGPT2 represents a different type of vascular regulator. Angiopoietins control vascular stability, angiogenesis, endothelial activation, and permeability through interactions with the Tie2 signaling system. ANGPT1 generally promotes vascular stabilization, whereas ANGPT2 often acts as a context-dependent antagonist or destabilizing factor, particularly during inflammation. In AD, ANGPT2 has recently been identified as a mediator that aggravates disease by promoting BBB dysfunction and neuroinflammation (49). Transcriptomic analyses of human AD brains revealed elevated ANGPT2 expression in endothelial cells, and experimental evidence supported its role in increasing vascular permeability, promoting neuroinflammatory responses, and worsening AD-related pathology. ANGPT2 is highly relevant to an inflammation-centered model of AD because it links endothelial activation with downstream immune and neuronal consequences. Increased ANGPT2 may destabilize endothelial junctions, increase BBB leakiness, and sensitize vessels to inflammatory cytokines. BBB disruption then allows peripheral inflammatory mediators to affect the CNS, while neuroinflammation further damages the vascular unit. In this way, ANGPT2 may act as both a vascular destabilizer and an inflammatory amplifier.

Together, DR6 and ANGPT2 highlight the complexity of endothelial signaling in AD. Some pathways, such as DR6-related protective signaling and Wnt/β-catenin activation, may help maintain BBB integrity, whereas others, such as ANGPT2-driven vascular destabilization and type I interferon activation, may promote leakiness and inflammation. The balance between protective and damaging endothelial programs may determine whether the BBB remains resilient or becomes a driver of AD progression.

### 3.6 BBB protection as therapeutic proof-of-concept

If BBB dysfunction actively contributes to AD progression, then preserving barrier integrity should provide therapeutic benefit. Although BBB-targeted therapies are not yet established in clinical AD treatment, recent experimental findings provide proof-of-concept that stabilizing the BBB can protect brain function. One example is inhibition of 15-hydroxyprostaglandin dehydrogenase (15-PGDH). 15-PGDH is an enzyme involved in prostaglandin metabolism and has been implicated in inflammatory and tissue-repair processes. Inhibition of 15-PGDH was shown to block BBB deterioration and protect mice from AD-related and traumatic brain injury-related pathology (107). The study reported that 15-PGDH is enriched in BBB-associated myeloid cells and becomes elevated in human and mouse models of AD and traumatic brain injury. Pharmacological inhibition of 15-PGDH preserved BBB integrity and supported brain health and cognition. This finding is significant for several reasons. First, it supports the idea that BBB deterioration is not merely a passive consequence of neurodegeneration but a modifiable process. Second, it highlights BBB-associated myeloid cells as important contributors to vascular-barrier deterioration. Third, it suggests that therapies aimed at preserving the vascular-immune interface may have broad relevance across neurodegenerative and injury-related conditions. In AD, such a strategy could complement anti-Aβ or anti-tau therapies by reducing the inflammatory and vascular environment that promotes neuronal injury.

**Figure 2:**
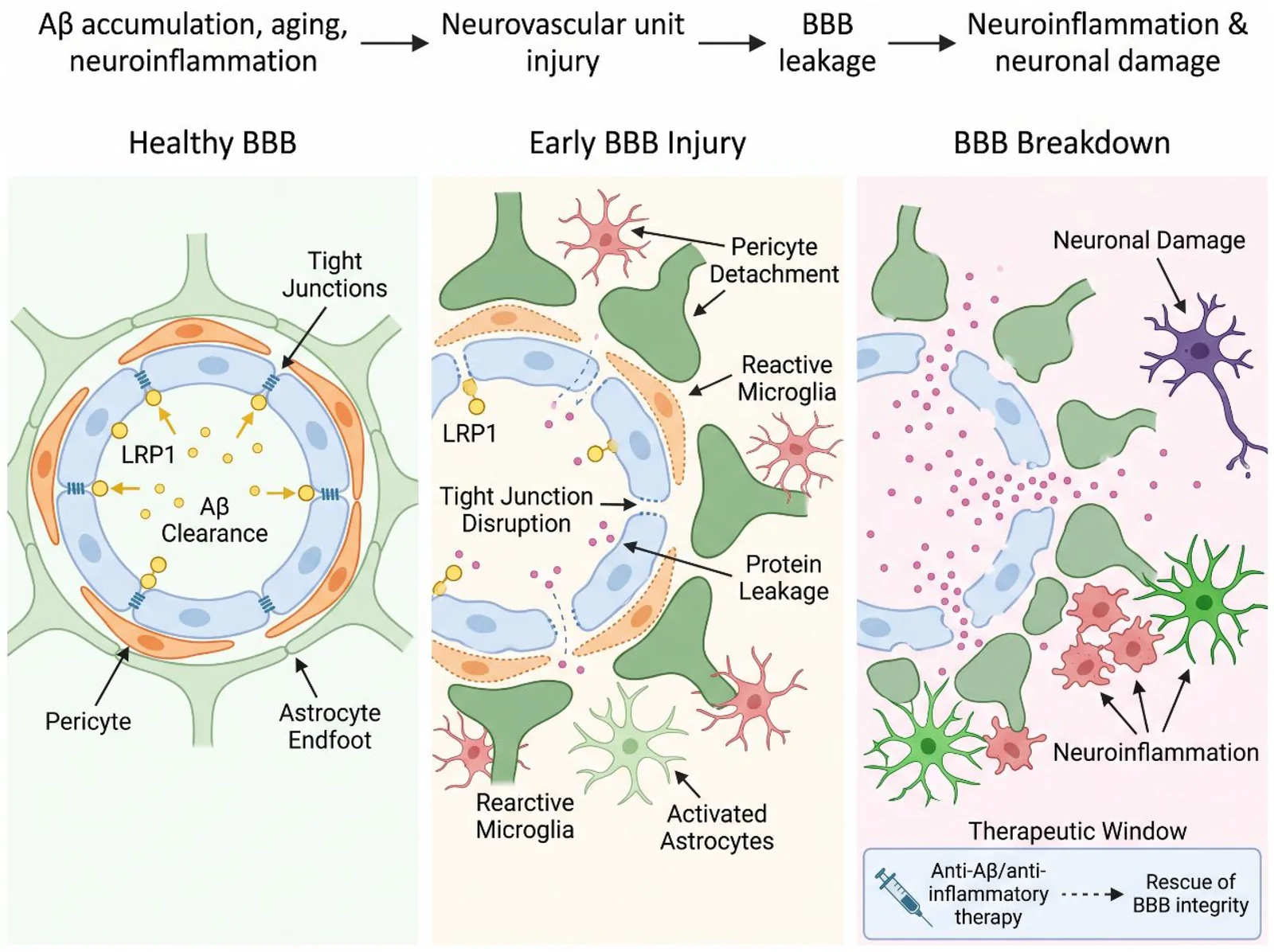
Molecular mechanisms driving BBB dysfunction in Alzheimer’s disease. Aβ injures pericytes through CD36/PINK1/Parkin-mediated mitophagy-dependent ferroptosis, whereas impaired Wnt/β-catenin signaling, type I interferon activation, vascular senescence, and ANGPT2 promote endothelial dysfunction and barrier leakage. Protective mechanisms, including DR6 signaling and 15-PGDH inhibition, may stabilize BBB integrity and reduce neuroinflammation.

However, BBB protection should currently be viewed as a therapeutic proof-of-concept rather than a mature clinical strategy (107). Several questions remain unresolved. It is unclear which patients would benefit most from BBB-targeted therapy, which biomarkers should be used to identify BBB dysfunction, and whether BBB stabilization alone is sufficient to slow AD progression. It is also important to determine whether BBB-targeted interventions should be applied early, before extensive neurodegeneration, or combined with amyloid-, tau-, or immune-directed therapies. Despite these uncertainties, the 15-PGDH study strengthens the rationale for treating the BBB as an active therapeutic target in AD (107). More broadly, BBB-protective strategies may include enhancing Wnt/β-catenin signaling, reducing endothelial inflammatory signaling (77), inhibiting ANGPT2-mediated vascular destabilization (49), preserving pericyte function and limiting ferroptotic injury, targeting vascular senescence (44), and modulating BBB-associated immune cells (107). These approaches share a common goal: to preserve the integrity of the neurovascular interface and prevent peripheral inflammatory signals from amplifying CNS pathology. **Figure 2** summarizes the major molecular mechanisms that disrupt BBB integrity in AD, including pericyte injury, endothelial inflammatory activation, vascular senescence, and loss of barrier-protective signaling. It also highlights BBB stabilization as a potential therapeutic strategy to limit neuroinflammation and AD progression. Although these mechanisms are discussed separately above, they converge on a limited set of neurovascular outcomes, including pericyte loss, endothelial junctional disruption, inflammatory activation, vascular senescence, and increased BBB permeability. Importantly, these pathways are not uniformly detrimental: Wnt/β-catenin and DR6-associated signaling appear to support barrier resilience, whereas excessive type I interferon signaling, ANGPT2 activation, vascular senescence, and Aβ-induced pericyte ferroptosis favor BBB disruption. The major pathways, involved cell types, supporting evidence, and therapeutic implications are compared in **Table 1**.

**Table 1:** Major molecular pathways involved in BBB dysfunction in Alzheimer’s disease.

Pathway/mechanism	Major cell type(s)	Effect on BBB	Supporting evidence	References	Therapeutic relevance
Aβ–CD36/PINK1/Parkin–mitophagy/ferroptosis	Pericytes	Promotes mitochondrial dysfunction, ferroptotic pericyte injury, and BBB destabilization	Aβ1–40 induced mitophagy-dependent ferroptosis in pericytes and promoted BBB destruction in experimental AD models	(55, 108)	Preservation of pericyte viability and inhibition of excessive ferroptotic injury may help maintain BBB integrity
Wnt/β-catenin signaling	Brain endothelial cells	Maintains endothelial barrier phenotype, tight junctions, and BBB stability	Activation of endothelial LRP6/Wnt/β-catenin signaling alleviated Aβ-induced BBB dysfunction	(67)	Endothelial-selective restoration of Wnt signaling represents a potential BBB-protective strategy
Type I interferon–IFNAR signaling	Brain endothelial cells	Promotes endothelial inflammatory activation, loss of junctional proteins, and increased permeability	IFN-β reduced VE-cadherin, occludin, and claudin-5 and increased endothelial leakiness; IFNAR depletion restored barrier integrity	(77)	Modulation of excessive endothelial type I interferon signaling may reduce inflammatory BBB injury
Vascular senescence/SASP	Endothelial cells and other neurovascular cells	Promotes age-related vascular leak, oxidative stress, inflammatory signaling, and impaired vascular repair	Vascular senescence and leakage were identified as early features of BBB breakdown in AD models	(44)	Senomorphic or vascular-rejuvenating approaches may reduce age-related BBB vulnerability, although clinical evidence remains limited
DR6-associated endothelial signaling	Brain endothelial cells	Supports BBB functional protein expression and endothelial resilience during Aβ stress	DR6 suppression aggravated Aβ-associated BBB malfunction, whereas DR6 overexpression improved barrier-related proteins	(106)	Modulation of DR6-associated protective signaling may enhance endothelial resilience
ANGPT2/Tie2 signaling	Brain endothelial cells	Destabilizes vascular integrity and promotes BBB permeability and neuroinflammation	ANGPT2 was increased in AD-associated endothelial cells and experimentally aggravated BBB dysfunction and neuroinflammation	(49)	ANGPT2 inhibition or restoration of ANGPT1/Tie2 balance may represent a vascular-stabilizing strategy
15-PGDH-associated BBB deterioration	BBB-associated myeloid cells/neurovascular interface	Contributes to BBB deterioration and impaired brain homeostasis	Pharmacological inhibition of 15-PGDH preserved BBB integrity and protected brain function in experimental models	(107)	15-PGDH inhibition provides proof-of-concept for pharmacological BBB stabilization

## 4 Multi-omics dissection of BBB and neurovascular unit alterations

### 4.1 Proteomic alterations in the brain and BBB during AD progression

Multi-omics approaches have provided new insight into BBB and neurovascular unit alterations in AD. Unlike single-pathway studies, omics analyses can capture coordinated changes across cell types, molecular networks, and disease stages. Proteomic studies are particularly useful because proteins directly reflect structural, enzymatic, signaling, and transport functions within the BBB and brain tissue. In the AppNL-G-F knock-in mouse model of AD, proteomic alterations were identified in both the brain and BBB compartments during Aβ accumulation and disease progression (109). This study supports the idea that BBB dysfunction is part of a broader proteomic remodeling process rather than an isolated abnormality of tight junctions alone. Changes in BBB-associated proteins may reflect altered transport, endothelial metabolism, inflammatory signaling, extracellular matrix remodeling, and vascular-glial communication.

The value of proteomic evidence lies in its ability to reveal system-level remodeling. In AD, BBB dysfunction likely involves multiple processes operating simultaneously, including endothelial stress, pericyte injury, basement membrane alteration, altered receptor-mediated transport, immune activation, and impaired clearance mechanisms (110–116). Proteomic alterations across the brain and BBB can therefore help identify molecular signatures that are not obvious from histological assessment alone. These signatures may also provide candidate biomarkers or therapeutic targets.

Proteomic studies also help connect Aβ pathology with vascular dysfunction. In AD models, Aβ accumulation may induce changes in proteins involved in barrier stability, inflammatory response, oxidative stress, lipid metabolism, and vascular repair. Conversely, BBB protein changes may influence Aβ handling by altering transport receptors, degradation pathways, or vascular clearance mechanisms. Thus, proteomic remodeling can be interpreted as both a consequence of AD pathology and a contributor to disease progression. Collectively, these proteomic findings support the view that BBB dysfunction in AD represents coordinated network-level remodeling rather than an isolated alteration of individual junctional proteins. Rather than focusing only on individual molecules, such as claudin-5 or occludin, it is important to consider how vascular, immune, metabolic, and glial protein networks are reorganized during AD. This perspective is especially useful for identifying vascular-inflammatory AD subtypes and for developing biomarker panels that integrate BBB integrity with neuroinflammatory activity.

### 4.2 Single-nucleus transcriptomics of impaired angiogenesis and BBB function

Single-nucleus transcriptomics has greatly improved understanding of cell-type-specific alterations in AD. Because the neurovascular unit contains multiple interacting cell types, bulk tissue analysis cannot easily distinguish whether observed changes originate from endothelial cells, pericytes, astrocytes, microglia, neurons, or other vascular-associated populations. Single-nucleus RNA sequencing allows researchers to resolve these cellular compartments and identify disease-associated transcriptional states. A single-nuclear transcriptomic study characterized mechanisms responsible for impaired angiogenesis and BBB function in AD (34). This work showed that endothelial cells are enriched for genes associated with AD susceptibility and that increased Aβ is associated with BBB-related alterations. Such findings suggest that endothelial cells are not passive bystanders in AD but may carry genetic and transcriptional programs that influence disease vulnerability.

Impaired angiogenesis is particularly relevant to AD progression. Healthy angiogenic and vascular repair responses are needed to maintain microvascular networks, support tissue perfusion, and adapt to metabolic stress (117–121). If endothelial cells in AD display defective angiogenic programs, the brain may become less capable of repairing vascular injury or responding to chronic hypoperfusion. This may worsen BBB dysfunction, reduce neurovascular coupling, and increase neuronal vulnerability. Single-nucleus transcriptomics also provides a framework for linking AD risk genes with vascular biology. Many AD-associated genes are traditionally interpreted in relation to microglia, lipid metabolism, or Aβ processing. However, endothelial enrichment of AD susceptibility genes suggests that vascular cells may also participate in genetic risk expression. This expands the mechanistic landscape of AD from neurons and glia to vascular and barrier-forming cells. Another important implication is that BBB dysfunction may differ across cell states and disease stages. Endothelial cells may transition from a homeostatic barrier phenotype to an inflammatory, senescent, angiogenesis-impaired, or permeability-prone phenotype. These transitions may be influenced by Aβ, tau-related stress, systemic inflammation, hypoxia, and aging. Therefore, single-nucleus studies can help identify when and how vascular cells shift toward disease-associated states.

### 4.3 Gliovascular transcriptional perturbations and cell–cell communication

The BBB is maintained by a multicellular gliovascular unit rather than by endothelial cells alone (89, 122). Endothelial cells, pericytes, astrocytes, microglia, basement membrane components, and perivascular macrophages continuously exchange signals that regulate vascular stability, barrier integrity, metabolic support, and immune responses (89, 122). Therefore, disruption of cell–cell communication within the gliovascular unit may be a central mechanism of BBB dysfunction in AD. Gliovascular transcriptional studies in AD have revealed extensive transcriptome changes across the gliovascular unit and have identified perturbed interactions among vascular and glial cell populations (35). In particular, altered pericyte–astrocyte communication, including disturbed SMAD3–VEGFA-related signaling, has been prioritized as a candidate mechanism linking neurovascular remodeling with BBB dysfunction. This finding is important because it moves beyond the idea that BBB breakdown is caused only by endothelial injury. Instead, it suggests that AD involves failure of coordinated multicellular support systems.

Pericyte–astrocyte signaling is essential for maintaining BBB integrity (123, 124). Pericytes support endothelial barrier phenotype, capillary stability, and vascular contractility, whereas astrocytes regulate water balance, metabolic exchange, vascular signaling, and inflammatory responses (123, 124). If pericyte-to-astrocyte or astrocyte-to-endothelial communication becomes dysfunctional, endothelial cells may lose critical trophic and structural support, leading to altered tight junction expression, increased permeability, impaired vascular repair, and enhanced inflammatory responsiveness (125). Gliovascular perturbations also provide a mechanistic link between BBB dysfunction and neuroinflammation in AD. Reactive astrocytes and activated microglia can release cytokines, chemokines, reactive oxygen species, complement-related factors, and matrix-remodeling enzymes, which may further compromise endothelial cells, pericytes, and BBB integrity (124, 126). In turn, BBB leakage can expose glial cells to plasma proteins and peripheral inflammatory signals, further amplifying their reactive states. Thus, gliovascular dysfunction may create a bidirectional loop between barrier breakdown and glial inflammation. Cell–cell communication analysis is particularly valuable because it identifies ligand–receptor interactions that may be targetable. For example, pathways involving TGF-β/SMAD, VEGF, angiopoietins, inflammatory cytokines, chemokines, and extracellular matrix regulators may determine whether the neurovascular unit remains stable or becomes dysfunctional (127–129). Future studies integrating spatial transcriptomics, proteomics, and functional BBB assays will be needed to validate these predicted interactions and determine their causal roles.

### 4.4 Choroid plexus and blood–CSF barrier abnormalities

Although the BBB is the most widely studied CNS barrier, the choroid plexus and blood–cerebrospinal fluid barrier are also highly relevant to AD. The choroid plexus is a vascularized epithelial structure located within the brain ventricles. It produces cerebrospinal fluid, regulates exchange between blood and CSF, contributes to immune surveillance, and secretes signaling molecules that influence brain homeostasis. Because the CSF communicates with multiple CNS compartments, choroid plexus dysfunction may affect inflammation, waste clearance, and neuroimmune communication. Recent work identified early transcriptional and cellular abnormalities in the choroid plexus of an AD mouse model (130). Using single-cell transcriptional profiling, this study showed that the choroid plexus undergoes molecular and cellular changes during early AD-like pathology. These abnormalities may include altered epithelial function, immune-related changes, lipid accumulation, and inflammatory signaling. Such findings broaden the concept of barrier dysfunction in AD beyond the classical endothelial BBB.

The blood–CSF barrier may influence AD progression through several mechanisms. Impaired choroid plexus epithelial function may alter CSF composition, reduce the clearance of neurotoxic metabolites, and disturb inflammatory mediator homeostasis (131, 132). Choroid plexus immune activation may also affect leukocyte trafficking and immune surveillance at the CNS border (133, 134). Changes in CSF production, turnover, and transport functions may further influence Aβ clearance and brain homeostasis (131, 135). Inflammatory signaling from the choroid plexus may communicate with ventricular and parenchymal compartments and contribute to glial activation; notably, early choroid plexus epithelial abnormalities in an AD mouse model were associated with altered macrophage signaling, lipid accumulation, and microglial activation (130). The choroid plexus is therefore particularly relevant to peripheral immune activation because the blood–CSF barrier functions as an immunologically active interface regulating immune-cell surveillance and trafficking (133, 136). If this interface becomes dysfunctional, peripheral inflammatory signals may gain greater access to CSF-associated compartments and modify CNS immune tone, thereby complementing BBB-mediated pathways of neuroinflammation (130, 137).

**Figure 3:**
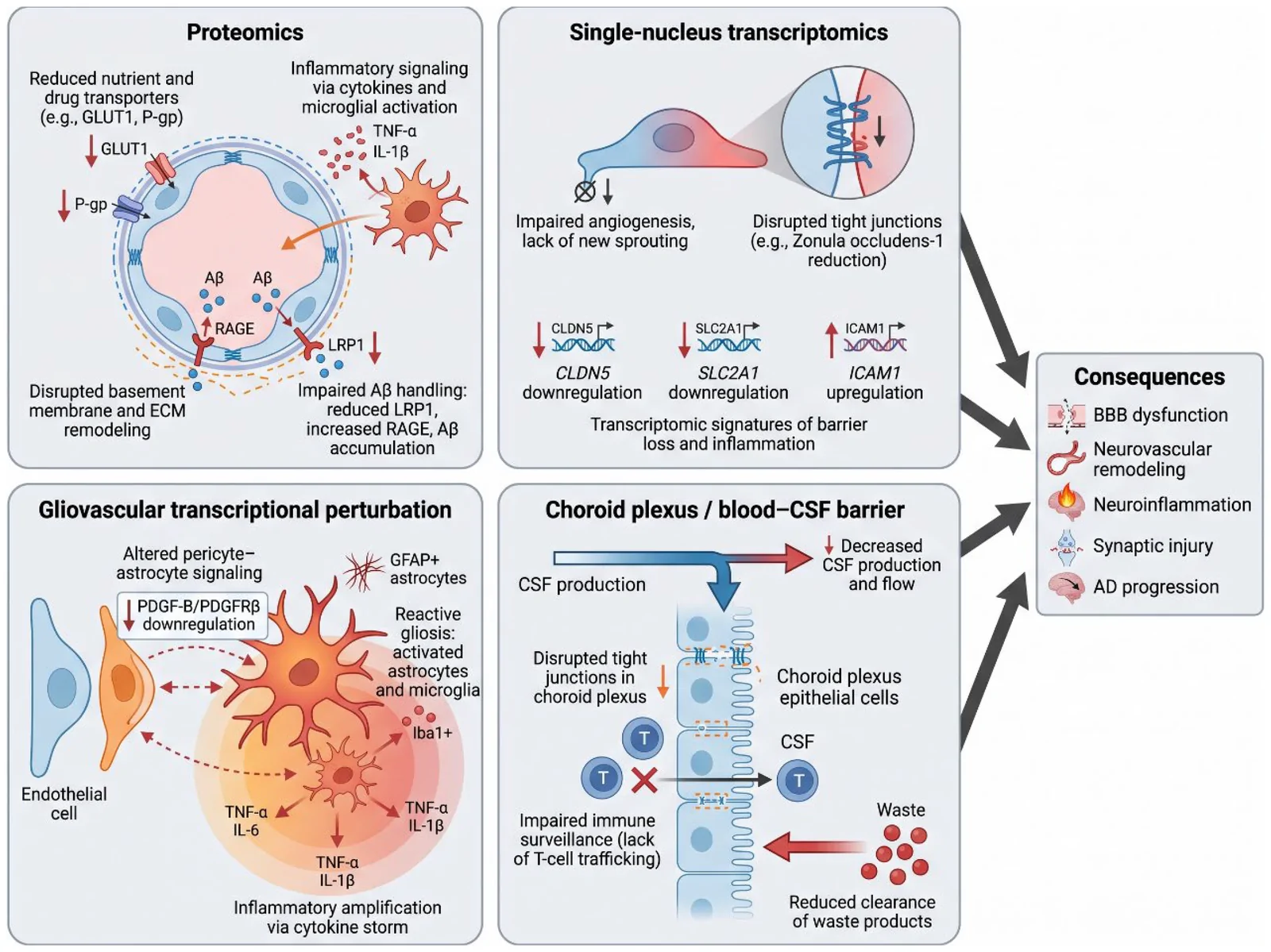
Multi-omics dissection of BBB and neurovascular-unit alterations in Alzheimer’s disease. Proteomic and single-nucleus transcriptomic studies reveal BBB and neurovascular-unit remodeling in AD, including altered transport, inflammatory signaling, impaired angiogenesis, endothelial state transition, and disrupted gliovascular communication. Choroid plexus and blood–CSF barrier abnormalities further indicate multi-barrier dysfunction, collectively promoting neuroinflammation, impaired clearance, synaptic injury, and AD progression.

Therefore, AD-related barrier dysfunction should be conceptualized as a multi-barrier process. The BBB, blood–CSF barrier, meningeal barriers, glymphatic system, and perivascular drainage pathways may all participate in regulating CNS exposure to peripheral signals and waste clearance. Including the choroid plexus in this review strengthens the argument that AD involves widespread disruption of CNS barrier and immune-interface biology, not simply focal endothelial leakage. Collectively, recent experimental, clinical, and multi-omics studies indicate that BBB dysfunction in AD involves not only endothelial tight-junction disruption but also pericyte injury, vascular senescence, inflammatory endothelial signaling, gliovascular remodeling, and blood–CSF barrier abnormalities. **Figure 3** summarizes how multi-omics studies reveal BBB dysfunction in AD as a multicellular and multi-barrier process. It highlights that endothelial alterations, impaired angiogenesis, disrupted gliovascular communication, and choroid plexus abnormalities converge on neurovascular remodeling, neuroinflammation, and disease progression.

## 5 Peripheral innate immune activation in AD

### 5.1 Peripheral monocytes as regulators of Aβ clearance and transport

Peripheral innate immunity is increasingly recognized as an active component of AD pathobiology. Among circulating innate immune cells, monocytes are particularly important because they can sense inflammatory cues, phagocytose pathological proteins, migrate toward injured tissues, differentiate into macrophage-like cells, and communicate with endothelial and perivascular compartments. In the context of AD, peripheral monocytes may influence disease progression through their capacity to bind, internalize, degrade, and transport Aβ. This expands the traditional view that Aβ clearance is mediated only by CNS-resident microglia, astrocytes, vascular transporters, and perivascular drainage pathways. Recent evidence indicates that peripheral monocytes participate in the clearance and transport of Aβ and that their Aβ-handling capacity correlates with AD progression (23). This finding is important for two reasons. First, it suggests that circulating monocytes are not merely passive inflammatory markers but may have functional roles in controlling peripheral and possibly CNS-related Aβ burden. Second, it implies that impaired monocyte function may contribute to disease progression by reducing the ability of the peripheral immune system to buffer or remove pathological Aβ species.

Peripheral monocytes may regulate Aβ homeostasis through several mechanisms (138–143). They can directly bind soluble or aggregated Aβ, internalize Aβ through phagocytic or receptor-mediated pathways, and degrade internalized material through lysosomal processing. They may also participate in Aβ transport by carrying Aβ-containing material in the circulation or by interacting with vascular endothelial cells at the BBB. In addition, monocytes can release cytokines, chemokines, proteases, reactive oxygen species, and lipid mediators that influence vascular inflammation and BBB integrity (144–149). Therefore, their role in AD may be dual: protective when they support Aβ clearance, but potentially harmful when they become chronically activated and promote systemic or vascular inflammation. This duality is central to understanding peripheral innate immunity in AD. Efficient monocyte-mediated Aβ clearance may help reduce pathological burden, whereas defective phagocytosis, excessive inflammatory activation, or abnormal monocyte–endothelial interactions may amplify neurovascular injury. In patients with AD progression, monocyte dysfunction may reflect immune aging, chronic antigenic stimulation, metabolic dysregulation, or sustained exposure to Aβ-related signals. These changes may reduce clearance capacity while increasing inflammatory output, thereby shifting monocytes from a protective to a disease-promoting state. Peripheral monocytes may also serve as accessible biomarkers. Because blood samples can be repeatedly obtained, monocyte Aβ-handling capacity, inflammatory phenotype, surface receptor expression, and transcriptomic signatures may help monitor disease progression or identify patients with a stronger vascular-inflammatory component. In this sense, monocytes are both functional contributors to AD biology and potential translational biomarkers for patient stratification. Importantly, the net effect of peripheral monocytes is unlikely to be uniformly protective or detrimental, but may depend on disease stage, monocyte functional phenotype, and the integrity of the BBB and perivascular immune interface.

### 5.2 Plasma Aβ aggregates and monocyte activation in mild cognitive impairment

Mild cognitive impairment represents a prodromal stage in which pathological processes associated with AD may already be active before the development of advanced dementia. Identifying immune changes at this stage is particularly important because early inflammatory activation may contribute to disease progression and may also provide a window for intervention. Peripheral immune activation in MCI suggests that AD-related pathology is not confined to the brain even during early clinical phases. A key study showed that Aβ aggregates in plasma can activate peripheral monocytes in individuals with mild cognitive impairment (24). This finding provides direct support for the concept that circulating Aβ species can act as immune-active ligands. Rather than being inert by-products of brain amyloidosis, plasma Aβ aggregates may be detected by innate immune cells and trigger inflammatory responses. This may help explain why peripheral immune alterations can be observed before overt dementia.

Monocyte activation by plasma Aβ aggregates may have several biological consequences. Aβ aggregates can alter monocyte activation and phagocytic behavior during the MCI stage, while Aβ-exposed monocytes are also capable of producing inflammatory cytokines and chemokines and modifying their interactions with vascular endothelium (150). These activated monocytes may influence the BBB through soluble inflammatory mediators or direct endothelial adhesion. Consistent with this possibility, Aβ can promote endothelial inflammatory activation and increase adhesion-related signaling, including VCAM-1 expression (151), and experimental BBB models have shown enhanced monocyte adhesion and transendothelial migration in response to Aβ (152, 153). If BBB integrity is already compromised by aging, vascular injury, or endothelial inflammation, peripheral immune activation may have greater consequences for CNS inflammatory signaling. Clinical biomarker evidence further indicates that BBB-related markers modulate the associations between peripheral immune abnormalities and AD pathology, cerebral atrophy, and cognition (17). Thus, BBB dysfunction may act as an important contextual factor determining how strongly peripheral monocyte activation influences the brain.

In early disease, this interaction may already be relevant. Large plasma Aβ aggregates have been identified in individuals with MCI and are associated with altered CD18-high monocyte populations, supporting peripheral innate immune engagement during prodromal disease. Over time, a feed-forward interaction may develop in which Aβ-related signals activate peripheral monocytes, vascular inflammation promotes BBB dysfunction, and impaired barrier integrity increases CNS exposure to peripheral inflammatory mediators (17, 151). The MCI setting also has potential biomarker implications. Because plasma Aβ aggregate-associated monocyte alterations can be detected before advanced dementia, combined assessment of circulating Aβ species and monocyte functional states may help characterize individuals with early immune involvement. In parallel, monocyte Aβ-binding, clearance, and transport capacity correlate with AD progression and have been proposed as potential blood-based biomarkers for disease monitoring. Such measures may complement established amyloid, tau, and neurodegeneration biomarkers by capturing the peripheral immune component of AD. However, whether these markers predict longitudinal cognitive decline, BBB deterioration, or treatment response remains to be established.

### 5.3 Peripheral monocyte-derived cells in AD mouse models

Animal models provide important mechanistic evidence for the participation of peripheral monocyte-derived cells in AD pathology. Although microglia are the dominant resident myeloid cells of the brain parenchyma, peripheral monocytes can enter CNS border regions and, under certain pathological conditions, may access brain parenchyma or plaque-associated niches. The extent, phenotype, and function of these monocyte-derived cells remain context-dependent and may vary according to disease stage, BBB integrity, chemokine signaling, and experimental model. In a mouse model of AD, peripheral monocyte-derived cells were shown to counter amyloid plaque pathogenesis. These cells invaded the brain parenchyma, targeted amyloid plaques, and contributed to reduced plaque load. This study is important because it challenges a purely harmful view of peripheral immune-cell entry into the AD brain. Instead, it suggests that recruited monocyte-derived cells may perform beneficial phagocytic or plaque-modulating functions under specific conditions.

The protective role of monocyte-derived cells may reflect functional differences between recruited peripheral myeloid cells and resident microglia. During chronic AD pathology, microglia may become metabolically stressed, exhausted, or functionally constrained by the plaque microenvironment (154–157). Peripheral monocytes may retain substantial phagocytic and remodeling capacity after recruitment. Once recruited to plaque-associated regions, monocyte-derived cells can engulf Aβ and modify plaque pathology; experimental evidence indicates that peripheral monocyte-derived cells can enter the AD brain and counter amyloid plaque pathogenesis (158). Chemokine-dependent recruitment, particularly involving the CCL2/CCR2 axis, may facilitate the accumulation of peripheral mononuclear phagocytes at vascular and parenchymal sites of Aβ deposition (159). However, recruitment of monocyte-derived cells also reflects remodeling of CNS barrier and perivascular immune interfaces. In human AD hippocampus, CD163-positive monocyte-derived cells were detected in the parenchyma and amyloid plaques, often in proximity to blood vessels, and their infiltration was associated with endothelial activation and potentially impaired BBB integrity (160). Importantly, the functional consequences of such recruitment appear to be context dependent. Beneficial effects may predominate when recruited cells retain efficient Aβ-clearing activity, whereas excessive or dysregulated recruitment into an inflamed neurovascular environment may contribute to inflammatory and vascular injury (158, 160). The net effect is therefore likely to depend on monocyte phenotype, timing of recruitment, chemokine signaling, BBB integrity, and the local plaque-associated microenvironment. Therapeutically, this argues against indiscriminate suppression of peripheral monocyte recruitment and instead favors strategies that preserve or enhance Aβ-clearing functions while limiting inflammatory neurovascular damage.

### 5.4 Human evidence of monocyte-derived cell invasion in AD brain tissue

Although animal studies are valuable, human pathological evidence is essential for establishing translational relevance. Human AD is more heterogeneous than mouse models and is influenced by aging, vascular disease, immune senescence, genetic background, metabolic disorders, and comorbid inflammation. Therefore, demonstrating monocyte-derived cell involvement in human AD tissue strengthens the argument that peripheral innate immunity participates in disease biology. Human hippocampal evidence showed that monocyte-derived cells invade the brain parenchyma and amyloid plaques in AD (160). This finding provides a direct translational bridge from experimental models to human pathology. The hippocampus is a critical region for memory and is among the most vulnerable areas in AD. The presence of monocyte-derived cells in hippocampal parenchyma and amyloid plaque regions suggests that peripheral myeloid cells may access disease-relevant brain compartments and interact with core AD lesions. This evidence has several implications. First, it supports the concept that the AD brain is not immunologically isolated from the periphery. Even if infiltration is limited or regionally restricted, peripheral monocyte-derived cells can be found in close association with amyloid pathology. Second, it suggests that amyloid plaques may serve as focal sites for myeloid recruitment, where local chemokines, complement activation, vascular changes, and glial signals create an immune-attracting microenvironment. Third, it reinforces the idea that BBB and perivascular changes may enable or regulate peripheral immune-cell access.

**Figure 4:**
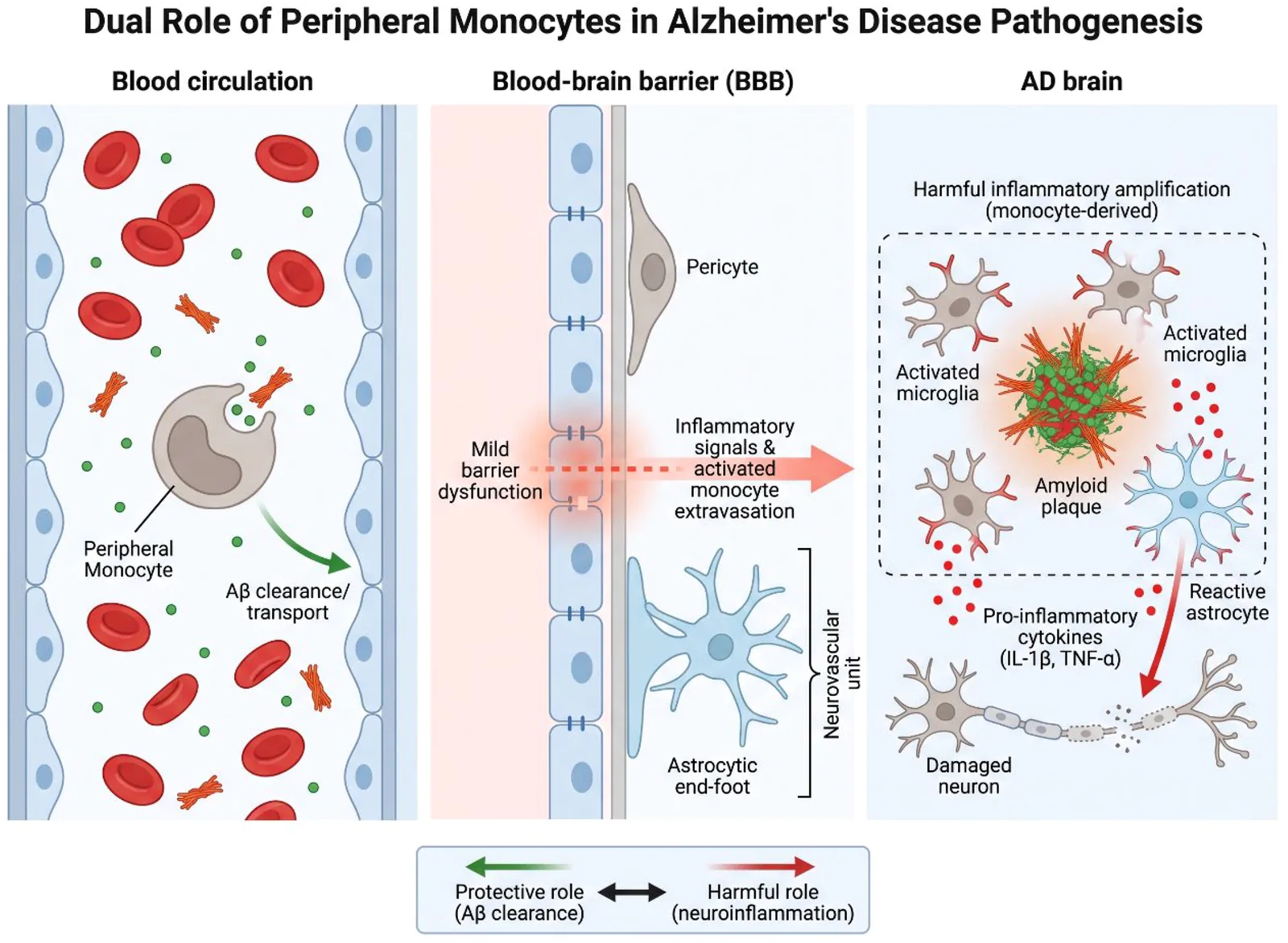
Peripheral monocytes in Alzheimer’s disease. Peripheral monocytes bind and transport circulating Aβ, supporting Aβ clearance. In MCI or AD, plasma Aβ aggregates activate monocytes and inflammatory signals. BBB dysfunction enables monocyte–neurovascular communication and recruitment toward amyloid plaques, where monocyte-derived cells may either promote plaque clearance or amplify glial activation, neuroinflammation, synaptic injury, and neuronal damage.

Human evidence also complicates the interpretation of monocyte-derived cells (161). Their presence near amyloid plaques does not automatically mean they are harmful or protective. They may participate in Aβ phagocytosis, antigen presentation, cytokine production, tissue remodeling, or vascular inflammation. Their phenotype may differ across patients and disease stages. Some may resemble macrophage-like phagocytes, whereas others may display inflammatory, antigen-presenting, or dysfunctional states. Therefore, future studies using single-cell transcriptomics, spatial profiling, lineage-informed markers, and functional assays are needed to define their roles more precisely. Human evidence of monocyte-derived cell invasion therefore provides direct pathological support for an interaction between peripheral innate immunity and AD lesions at the tissue level. Together with studies showing peripheral monocyte Aβ transport and plasma Aβ aggregate-induced activation, these findings support a continuous immune axis: circulating Aβ species activate monocytes, monocyte functional capacity correlates with disease progression, monocyte-derived cells can access amyloid pathology, and BBB/perivascular remodeling may regulate this communication. **Figure 4** illustrates how peripheral monocytes interact with circulating Aβ, the BBB, and amyloid pathology in AD. It highlights their dual role as Aβ-clearing immune cells and potential drivers of neurovascular inflammation and neuronal injury.

### 5.5 Context-dependent roles of peripheral monocytes: disease stage, phenotype, and BBB integrity

The functional impact of peripheral monocytes may vary across the AD continuum. During prodromal or early disease, circulating Aβ species can already activate monocytes, as demonstrated in individuals with mild cognitive impairment. At this stage, monocyte activation may represent an early systemic response to emerging Aβ pathology, but whether this response is predominantly protective or inflammatory is likely to depend on the balance between Aβ clearance capacity and inflammatory activation. Experimental studies further suggest that recruited monocyte-derived cells can counter amyloid plaque pathogenesis under selected conditions, supporting a potentially beneficial role when endogenous CNS clearance mechanisms are insufficient (158). In contrast, persistent or dysregulated monocyte responses in a vascularly compromised environment may favor inflammatory signaling and secondary neurovascular injury. For example, peripheral monocyte recruitment has been associated with increased vascular permeability and microhemorrhage-associated vascular inflammation in experimental AD models.

Monocyte phenotype is likely another major determinant of functional outcome. Monocytes with preserved phagocytic, lysosomal, and Aβ-processing capacity may contribute to peripheral Aβ clearance, consistent with the association between monocyte Aβ-handling capacity and AD progression. Experimental enhancement of monocyte Aβ uptake and intracellular processing also reduced amyloid deposition and AD-like pathology in APP/PS1 mice. By contrast, dysfunctional monocyte states may impair Aβ clearance or promote inflammatory injury. Notably, monocyte-derived cystatin F was increased in AD and inhibited monocyte Aβ phagocytosis, while enhancing amyloid deposition and cognitive deficits in experimental models. These findings illustrate that the biological effect of peripheral monocytes is determined not simply by their presence or recruitment, but by their activation state, phagocytic competence, metabolic status, and differentiation trajectory.

BBB integrity may further determine whether peripheral monocyte responses remain predominantly systemic, accumulate within perivascular compartments, or extend into the brain parenchyma. When BBB function is relatively preserved, monocytes may influence AD mainly through peripheral Aβ handling and soluble immune mediators. In contrast, endothelial activation, altered chemokine signaling, or barrier disruption may facilitate monocyte adhesion and transendothelial migration. The CXCL1/CXCR2 axis, for example, has been shown to promote Aβ-induced transendothelial migration of monocytes and to disrupt endothelial barrier properties. Human pathological evidence demonstrating monocyte-derived cells within hippocampal parenchyma and amyloid plaques further supports the relevance of peripheral myeloid recruitment in AD (160). Importantly, greater monocyte access to the CNS is not inherently beneficial, because the consequences of recruitment appear to depend on the vascular and inflammatory microenvironment (158, 160). Clinical biomarker evidence further indicates that BBB-related markers modify the associations between peripheral immune abnormalities and AD pathology, brain atrophy, and cognition (17). BBB status may therefore act as a gatekeeper that influences whether peripheral monocyte activity contributes predominantly to Aβ clearance or inflammatory amplification. Thus, peripheral monocytes should not be classified as uniformly protective or pathogenic in AD; their net effect likely emerges from the interaction among disease stage, cellular phenotype, recruitment context, and BBB integrity.

## 6 Peripheral adaptive immune changes in AD

### 6.1 T-cell exhaustion, amyloid accumulation, and cognitive status

Adaptive immunity is increasingly implicated in AD, particularly through alterations in T-cell function. T cells are not traditionally considered central drivers of AD, but growing evidence suggests that systemic immune aging, chronic inflammation, antigenic stimulation, and altered CNS barrier function may reshape T-cell responses during disease progression (162–166). Among these changes, T-cell exhaustion is especially relevant because it reflects chronic immune activation and impaired effector function. T-cell exhaustion has been associated with cognitive status and amyloid accumulation in AD (30). Exhausted T cells are typically characterized by altered cytokine production, reduced proliferative capacity, impaired effector responses, and increased expression of inhibitory receptors. In chronic infections and cancer, exhaustion develops after persistent antigen exposure. In AD, a similar exhaustion-like phenotype may indicate prolonged immune stimulation driven by amyloid pathology, inflammatory mediators, aging-related immune dysregulation, or repeated exposure to CNS-derived antigens.

The association between T-cell exhaustion, amyloid accumulation, and cognition suggests that adaptive immune dysfunction is not merely a peripheral epiphenomenon. It may reflect the systemic immune consequences of chronic AD pathology and may also contribute to disease progression. Exhausted or dysregulated T cells could alter cytokine balance, impair immune surveillance, influence monocyte and macrophage function, and interact with vascular endothelium (167–171). In addition, if BBB integrity is compromised, T-cell-derived cytokines or T-cell trafficking signals may more strongly influence CNS inflammation. T-cell exhaustion may also be linked to immune aging. Older adults often show thymic involution, reduced naive T-cell output, expansion of memory and senescent T-cell populations, chronic low-grade inflammation, and reduced immune flexibility. AD may intensify or reshape these age-related immune changes. In this context, exhausted T-cell signatures may represent a combined effect of aging, chronic systemic inflammation, and AD-associated proteinopathy. Beyond exhaustion, different T-cell subsets may exert distinct effects in AD. CD8^+^ effector-memory and TEMRA-like cells have been associated with cognitive impairment and chronic antigenic stimulation (31, 172), whereas brain-resident CD8^+^ T cells may show context-dependent protective functions (165). CD4^+^ regulatory T cells may also exert protective effects by restraining excessive inflammation and modulating microglial responses, as supported by experimental studies using Aβ-specific Tregs (166, 173). These findings indicate that T-cell involvement in AD is heterogeneous and cannot be explained by exhaustion alone.

From a mechanistic perspective, T-cell dysfunction could influence AD through several routes. Peripheral T cells may produce cytokines that affect endothelial activation, BBB permeability, and glial responses. They may regulate monocyte activation and differentiation through cytokine networks. In settings of barrier dysfunction, T cells or their soluble mediators may access CNS border regions, CSF compartments, or perivascular spaces. Even without extensive parenchymal infiltration, altered adaptive immune tone may shape the inflammatory environment surrounding the BBB. Therefore, T-cell exhaustion should be interpreted as part of a broader peripheral immune remodeling process in AD. It may serve as a biomarker of chronic inflammatory burden, amyloid-associated immune stimulation, or reduced immune resilience. Future work should determine whether T-cell exhaustion is a driver of cognitive decline, a consequence of AD pathology, or a marker of systemic immune aging that modifies disease trajectory.

### 6.2 Adaptive immune signatures across AD progression

Beyond T-cell exhaustion, broader adaptive immune changes have been associated with clinical progression of AD (31). These findings indicate that AD progression involves coordinated changes in immune-cell compartments rather than isolated alterations in a single cell type. Adaptive immune changes may include shifts in T-cell subsets, B-cell populations, activation states, clonal expansion, cytokine profiles, and immune aging signatures. The involvement of adaptive immunity is biologically plausible. AD is a chronic disease that develops over years or decades, during which persistent amyloid and tau pathology, glial activation, vascular dysfunction, and systemic inflammatory changes may repeatedly stimulate immune networks. Peripheral adaptive immune cells may respond to CNS-derived antigens, circulating Aβ species, damage-associated signals, microbial or metabolic stimuli, or age-related inflammatory cues. As these signals accumulate, adaptive immunity may shift from a flexible surveillance state toward chronic activation, exhaustion, senescence, or dysregulated memory responses.

T cells may contribute to AD progression through cytokine-mediated effects on endothelial cells, monocytes, microglia, and astrocytes (174–176). Pro-inflammatory T-cell responses could enhance BBB activation, increase leukocyte adhesion, and amplify glial inflammation. Regulatory T-cell dysfunction could reduce control over systemic and CNS-associated inflammation. Senescent or exhausted T-cell populations may produce inflammatory mediators while losing effective regulatory or protective functions. B cells may also contribute through antibody production, antigen presentation, cytokine release, and interactions with T-cell responses, although their precise role in AD remains less clearly defined. Adaptive immune signatures may also differ across disease stages. In early stages, subtle immune activation may reflect systemic responses to emerging amyloid pathology or vascular stress. During progression, immune remodeling may become more pronounced and may correlate with cognitive decline, neurodegeneration, or biomarker-defined pathology. In advanced disease, immune exhaustion or senescence may dominate, potentially reducing effective clearance responses while maintaining chronic inflammatory signaling. Recent evidence further suggests that these changes may be stage dependent. Effector and activated CD8^+^ T-cell responses may become detectable during early or prodromal stages (31, 169), whereas more differentiated, clonally expanded, tissue-resident, senescent, or exhaustion-associated populations may become more prominent as pathology progresses (163). Importantly, the functional consequences of these populations may also change over time, because some CD8^+^ T-cell states appear detrimental during early amyloid pathology but protective or compensatory at later stages (163). Thus, T-cell remodeling across the AD continuum is likely qualitative as well as quantitative.

These observations have important implications for AD classification. Classical biomarker frameworks focus on amyloid, tau, and neurodegeneration. However, adaptive immune signatures may identify patients with a stronger inflammatory or immune-aging component. Such patients may be more likely to show BBB dysfunction, vascular inflammation, or altered responses to immunomodulatory interventions. Therefore, integrating adaptive immune profiling with BBB biomarkers, Aβ/tau markers, neuroimaging, and cognitive measures may help define clinically meaningful AD subtypes. Therapeutically, adaptive immunity should be approached carefully. Broad immunosuppression may be harmful, especially in older adults, because immune surveillance and infection defense are already compromised. A more rational approach may involve identifying specific maladaptive immune programs, such as excessive endothelial-activating cytokine production, T-cell exhaustion, immune senescence, or abnormal T-cell–monocyte interactions. Targeting these specific programs may help reduce inflammatory damage while preserving protective immune functions.

### 6.3 BBB biomarkers as mediators between peripheral immunity and AD pathology

The strongest conceptual link between peripheral immune activation and AD pathology is the BBB. Peripheral immune alterations do not automatically influence the brain; their impact depends partly on how effectively immune signals can be transmitted across CNS barriers. BBB integrity may therefore determine whether systemic inflammation remains peripheral or becomes neuroinflammatory. This makes BBB biomarkers central to understanding the relationship between peripheral immunity and AD. Recent evidence showed that BBB-related biomarkers modulate the associations of peripheral immune biomarkers with AD pathology, cerebral atrophy, and cognitive function (17). This finding directly supports the premise of this review. It suggests that peripheral immunity may influence AD partly through BBB dysfunction, and that barrier integrity can shape how immune signals relate to brain pathology and clinical outcomes. This relationship can be understood through several mechanisms. First, BBB disruption may allow circulating cytokines, chemokines, complement components, plasma proteins, and other inflammatory mediators to enter the CNS or perivascular compartments. Second, endothelial activation may transmit inflammatory signals across the barrier without full structural leakage, by altering transporter expression, releasing cytokines, or activating perivascular glial cells. Third, BBB dysfunction may facilitate immune-cell adhesion, perivascular accumulation, or selective trafficking of monocytes and lymphocytes. Fourth, impaired BBB transport may reduce Aβ clearance and increase exposure of vascular and glial cells to toxic protein species. T-cell–BBB interactions are likely bidirectional (177). Activated T cells can release cytokines that enhance endothelial inflammatory signaling, alter junctional stability, and increase the expression of adhesion molecules and chemokines, thereby promoting further immune–endothelial interactions (14, 178). Conversely, endothelial activation and BBB dysfunction may facilitate T-cell adhesion, perivascular accumulation, and selective entry into CNS-associated compartments (177, 179). Experimental evidence demonstrating increased CD8^+^ T-cell infiltration in human AD brain tissue and AD mouse models, together with the identification of brain-resident CXCR6^+^PD-1^+^ CD8^+^ T cells, further supports a functional connection between adaptive immune remodeling and CNS barrier status (165, 171).

BBB biomarkers may therefore serve as biological mediators, moderators, or amplifiers. As mediators, they may lie on the causal pathway between peripheral immune activation and neurodegeneration. As moderators, they may determine which patients are more vulnerable to the effects of systemic inflammation. As amplifiers, they may intensify inflammatory feedback loops between the periphery and CNS. These roles are not mutually exclusive and may vary across disease stages. This concept is especially important for patient stratification. Two patients may have similar levels of peripheral inflammation, but the patient with greater BBB dysfunction may experience stronger CNS effects and faster cognitive decline. Conversely, preserved BBB integrity may buffer the brain against systemic immune activation. Therefore, combining peripheral immune markers with BBB biomarkers may provide more informative risk prediction than either category alone (180–182). Potential BBB-related biomarkers may include markers of endothelial injury, tight junction disruption, vascular permeability, basement membrane remodeling, pericyte dysfunction, and albumin leakage. When integrated with immune biomarkers such as monocyte activation markers, neutrophil-to-lymphocyte ratio, cytokines, chemokines, T-cell exhaustion markers, and adaptive immune signatures, they may help define a vascular-inflammatory AD phenotype.

**Figure 5:**
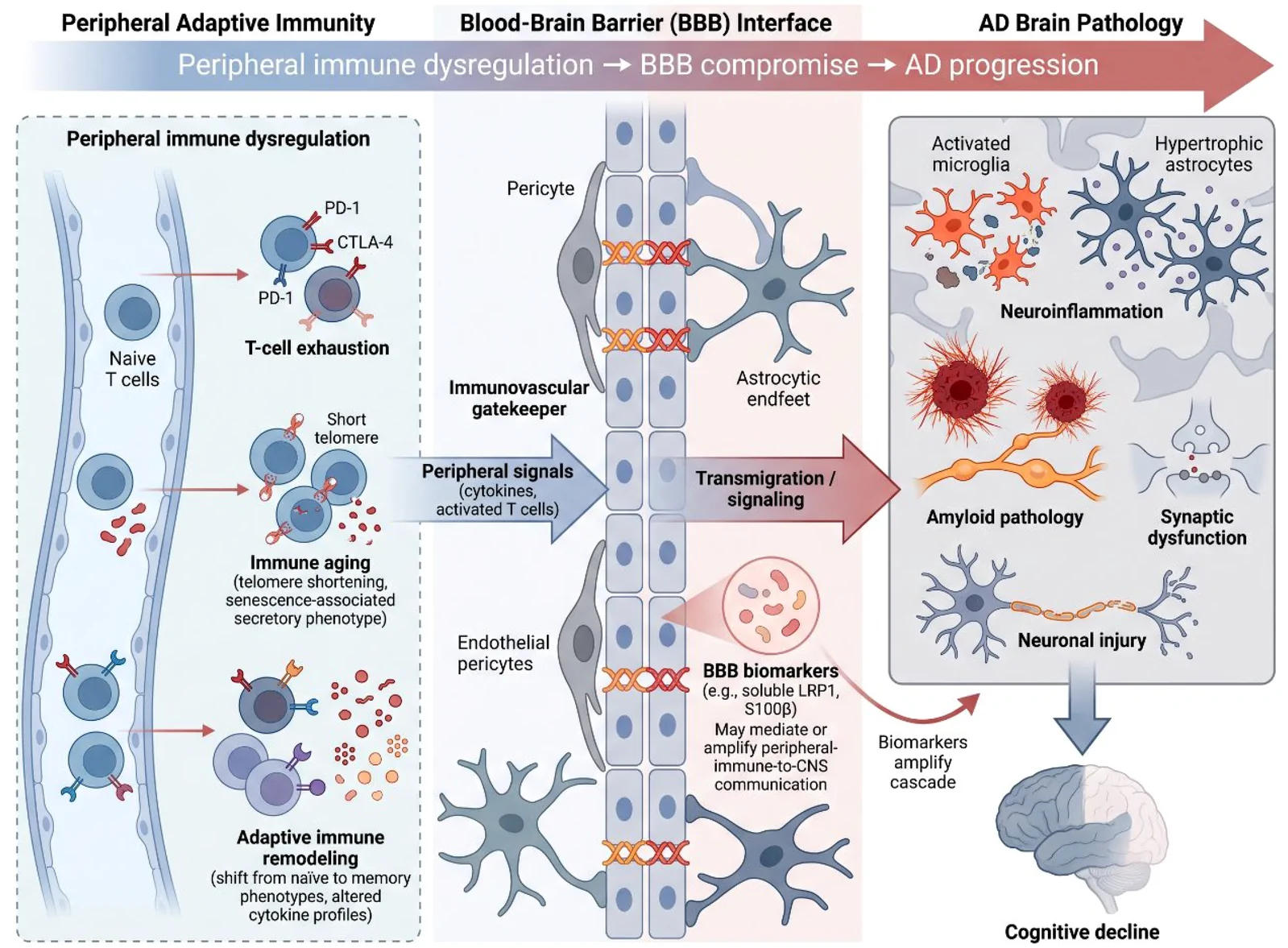
Peripheral adaptive immune remodeling in Alzheimer’s disease. Peripheral T-cell exhaustion, immune aging, and broader adaptive immune remodeling generate systemic inflammatory signals that interact with BBB dysfunction. As an immunovascular gatekeeper, the BBB modulates peripheral immune communication with the CNS, promoting glial activation, neuroinflammation, synaptic injury, neuronal damage, and cognitive decline in AD.

BBB biomarkers may provide an important biological bridge between peripheral immune dysregulation and CNS pathology, thereby integrating the vascular and immune dimensions of AD. The evidence suggests that peripheral immune abnormalities are clinically meaningful not simply because they occur in AD, but because their relationship with cognition and pathology may depend on BBB status. This supports an integrated model in which AD progression is shaped by the interaction among systemic immunity, vascular barrier dysfunction, glial activation, and neurodegeneration. In addition to CNS-resident glial activation, accumulating evidence suggests that peripheral innate and adaptive immune responses participate in AD progression. Key studies linking monocytes, monocyte-derived cells, T-cell exhaustion, adaptive immune remodeling, and BBB-related biomarkers to AD pathology are summarized in **Table 2**. **Figure 5** illustrates how peripheral adaptive immune dysregulation, including T-cell exhaustion and immune aging, may influence AD progression through the BBB interface. It highlights BBB dysfunction as a key mediator linking systemic immune remodeling with CNS neuroinflammation and cognitive decline.

**Table 2:** Peripheral innate and adaptive immune activation in Alzheimer’s disease.

References	Immune component	Model/sample	Main findings	Interpretation in the BBB–peripheral immune axis
(23)	Peripheral monocytes	Human AD-related samples	Peripheral monocytes participated in Aβ clearance and transport, and their Aβ-handling capacity correlated with AD progression.	Monocytes may function as both Aβ-clearance effectors and blood-based biomarkers of disease progression.
(24)	Plasma Aβ aggregates and monocytes	Mild cognitive impairment samples	Plasma Aβ aggregates activated peripheral monocytes in MCI.	Indicates that peripheral innate immune activation may occur during prodromal AD stages.
(158)	Monocyte-derived cells	AD mouse model	Peripheral monocyte-derived cells entered the brain and countered amyloid plaque pathogenesis.	Suggests recruited monocyte-derived cells may support plaque clearance under certain conditions.
(160)	Monocyte-derived cells in human brain	Human AD hippocampal tissue	Monocyte-derived cells were detected in brain parenchyma and amyloid plaques.	Provides human pathological evidence that peripheral myeloid cells can access AD-relevant brain regions.
(30)	T-cell exhaustion	Human AD-related immune profiling	T-cell exhaustion was associated with cognitive status and amyloid accumulation.	Links adaptive immune dysfunction with amyloid burden and clinical impairment.
(31)	Adaptive immune remodeling	Human AD progression cohort	Adaptive immune changes were associated with clinical progression of AD.	Supports the concept that systemic immune remodeling accompanies disease progression.
(17)	BBB biomarkers and peripheral immunity	Human biomarker cohort	BBB biomarkers modulated the associations between peripheral immune markers and AD pathology, atrophy, and cognition.	Directly supports BBB status as a mediator between peripheral immunity and CNS neurodegeneration.

## 7 Integrated inflammation-centered model: BBB dysfunction and peripheral immunity form a pathogenic loop

### 7.1 Initiation: Aβ, tau, aging, and vascular stress

The interaction between BBB dysfunction and peripheral immune activation in AD can be conceptualized as a self-reinforcing pathogenic loop. This loop is likely initiated by the convergence of Aβ deposition, tau pathology, aging-related vascular decline, metabolic stress, oxidative injury, and endothelial inflammatory activation. Although Aβ and tau are traditionally viewed as neuronal or synaptic pathologies, they also exert substantial effects on the neurovascular unit. Endothelial cells, pericytes, astrocytic endfeet, basement membrane components, and perivascular immune cells are all exposed to AD-related stressors and may participate in the earliest stages of vascular dysfunction. Aβ can directly injure vascular cells and weaken BBB integrity. In particular, Aβ-induced pericyte damage provides a mechanistic bridge between amyloid pathology and BBB breakdown. Pericytes are essential for endothelial stability, tight junction maintenance, suppression of excessive transcytosis, and capillary regulation. Aβ-induced activation of the CD36/PINK1/Parkin pathway can impair mitochondrial homeostasis and promote mitophagy-dependent ferroptosis in pericytes, leading to BBB destruction (55). Through this mechanism, Aβ pathology may compromise BBB integrity by targeting a key structural and functional support cell of the neurovascular unit.

Endothelial signaling is also altered during AD progression. The Wnt/β-catenin pathway is a central BBB-protective program that maintains endothelial barrier identity, tight junction integrity, and vascular stability. In AD models, activation of Wnt/β-catenin signaling can mitigate BBB dysfunction, suggesting that impaired endothelial protective signaling may contribute to barrier failure and that its restoration may stabilize the BBB (67). Similarly, endothelial DR6 has been implicated in BBB malfunction in AD, with evidence suggesting that DR6-related signaling may influence BBB functional protein expression and endothelial responses to Aβ stress (106). These findings indicate that BBB dysfunction is not only caused by direct toxic injury but also by dysregulation of endogenous vascular-protective pathways. Aging further primes the neurovascular unit for dysfunction. Vascular senescence and leak appear to be early features of BBB breakdown in AD models, supporting the idea that senescent endothelial or perivascular cells may create a permissive environment for barrier failure (44). Senescent vascular cells can secrete inflammatory cytokines, chemokines, extracellular matrix-remodeling enzymes, and reactive oxygen species, collectively forming a senescence-associated inflammatory microenvironment. This state weakens BBB homeostasis and increases susceptibility to Aβ, tau, hypoxia, metabolic dysfunction, and systemic inflammation. ANGPT2 represents another endothelial mediator that links vascular stress with inflammation. Increased ANGPT2 can destabilize endothelial junctions, promote vascular permeability, and enhance inflammatory responses. Recent evidence indicates that ANGPT2 aggravates AD by promoting BBB dysfunction and neuroinflammation (49). This supports a model in which endothelial destabilization is not merely a local vascular abnormality but a driver of broader neuroinflammatory progression.

Therefore, the initiation phase of the BBB–peripheral immune loop involves multiple converging insults. Aβ damages pericytes and vascular cells; aging and senescence weaken endothelial resilience; protective pathways such as Wnt/β-catenin may become insufficient; and inflammatory vascular mediators such as ANGPT2 promote barrier instability. These processes impair tight junction integrity, alter vascular signaling, increase permeability, and reduce the ability of the BBB to maintain CNS immune and metabolic homeostasis.

### 7.2 Amplification: BBB leakage and immune mediator entry

Once BBB integrity is compromised, the neurovascular interface shifts from a protective barrier to an amplifier of inflammation. BBB leakage can allow plasma proteins, inflammatory cytokines, chemokines, complement components, coagulation-related molecules, and other blood-derived factors to enter the perivascular space or brain parenchyma. Even modest increases in permeability may be sufficient to alter the local environment surrounding endothelial cells, pericytes, astrocytic endfeet, microglia, and neurons. In this way, BBB dysfunction can translate peripheral inflammatory states into CNS inflammatory responses. Inflammatory endothelial signaling plays a key role in this amplification phase. Type I interferon signaling has been shown to contribute to brain endothelial barrier dysfunction in an experimental model of AD (77). IFN-related signaling can reduce the expression of junctional proteins such as VE-cadherin, occludin, and claudin-5, thereby weakening endothelial adhesion and tight junction integrity. This mechanism demonstrates how inflammatory cytokine signaling can directly increase endothelial leakiness and promote barrier dysfunction.

BBB permeability is also associated with distinct neuroinflammatory profiles in AD, indicating that barrier dysfunction may shape inflammatory heterogeneity across patients (16). Patients with greater BBB permeability may show different patterns of inflammatory biomarkers, suggesting that BBB disruption defines a biologically meaningful vascular-inflammatory state. This is important because AD is not a uniform disease; the contribution of vascular dysfunction and inflammation may vary across individuals. BBB permeability may therefore help identify patients in whom peripheral immune signals have a stronger impact on CNS pathology. At the tissue level, BBB leakage can activate CNS-resident glial cells. Plasma-derived proteins and inflammatory mediators can stimulate microglia and astrocytes, leading to cytokine release, oxidative stress, complement activation, synaptic pruning, and extracellular matrix remodeling. Reactive astrocytes may further impair vascular function by altering astrocytic endfeet, water transport, and metabolic support. Activated microglia can release inflammatory mediators and reactive oxygen species that damage neurons and vascular cells. Thus, barrier leakage and glial activation can reinforce each other.

Gliovascular transcriptional studies support this multicellular amplification model. Perturbations in endothelial, pericyte, astrocyte, and other vascular-associated cell populations reveal disrupted cell–cell communication within the gliovascular unit (35). These findings suggest that BBB dysfunction in AD is not limited to endothelial tight junction loss but involves breakdown of coordinated support among vascular and glial cells. Once these interactions are disturbed, inflammatory signals can propagate more easily across the neurovascular unit. BBB biomarkers further connect peripheral immunity with AD outcomes. Evidence that BBB-related biomarkers modulate the associations between peripheral immune biomarkers and AD pathology, brain atrophy, and cognitive function supports the idea that BBB dysfunction acts as a biological gatekeeper between systemic inflammation and CNS injury (17). In this model, peripheral inflammation may be more damaging when BBB integrity is compromised. Conversely, preserved BBB function may buffer the brain against systemic immune activation.

Therefore, BBB leakage amplifies AD progression through both structural and signaling mechanisms. Structurally, it permits abnormal exposure of the CNS to blood-derived molecules. Functionally, it activates endothelial and glial inflammatory programs that sustain chronic neuroinflammation. This amplification stage is central to the proposed model because it explains how local AD pathology and systemic immune activation become integrated into a single pathogenic circuit.

### 7.3 Peripheral monocytes: protective clearance versus inflammatory invasion

Peripheral monocytes occupy a dual position in AD pathophysiology. On one hand, they may contribute to Aβ clearance, plaque-associated responses, and immune surveillance. On the other hand, their activation and recruitment may reflect systemic inflammation, vascular dysfunction, and BBB remodeling. This duality makes monocytes a key cellular component of the BBB–peripheral immune axis. Peripheral monocytes can bind, internalize, transport, and clear Aβ. Their capacity to handle Aβ correlates with AD progression, suggesting that monocyte function is biologically relevant to disease trajectory (23). When monocytes retain effective phagocytic and degradative capacity, they may help reduce Aβ burden or limit toxic circulating Aβ species. However, impaired monocyte Aβ handling may contribute to pathological accumulation, immune activation, or vascular exposure to Aβ aggregates.

Peripheral monocyte activation may occur early in the disease continuum. Plasma Aβ aggregates can activate peripheral monocytes in mild cognitive impairment, indicating that innate immune activation may begin before advanced dementia (24). This finding supports the idea that peripheral immunity can sense AD-related protein pathology outside the CNS. Activated monocytes may produce inflammatory cytokines, upregulate adhesion molecules, alter phagocytic programs, and interact more strongly with the vascular endothelium. These changes may increase endothelial activation and contribute to BBB dysfunction. Animal studies further show that peripheral monocyte-derived cells can enter the AD brain and interact with amyloid plaques. In an AD mouse model, peripheral monocyte-derived cells were reported to counter amyloid plaque pathogenesis (158). This suggests that recruited monocyte-derived cells can have beneficial functions, particularly in plaque clearance or modification of plaque-associated pathology. Therefore, peripheral immune-cell access to the CNS should not be interpreted as uniformly harmful.

However, the entry of monocyte-derived cells also reflects changes in CNS barrier and perivascular immune regulation. Under physiological conditions, the BBB restricts uncontrolled immune-cell infiltration. Recruitment of peripheral monocytes into brain parenchyma or plaque-associated regions implies endothelial activation, chemokine production, altered perivascular niches, or barrier remodeling. If this process becomes excessive or chronically inflammatory, recruited cells may release cytokines, proteases, reactive oxygen species, and other mediators that damage the neurovascular unit and surrounding neurons. Human evidence supports the translational relevance of this mechanism. Monocyte-derived cells have been identified in brain parenchyma and amyloid plaques in the hippocampus of human AD cases (160). This finding bridges animal models and human disease, indicating that peripheral myeloid cells can access AD-relevant brain regions and interact with amyloid pathology. Their exact role may vary depending on disease stage, local microenvironment, and activation phenotype.

Within the integrated BBB–peripheral immune model, monocyte function is best understood as context dependent rather than intrinsically protective or pathogenic. Early or functionally competent monocyte responses may support Aβ clearance, whereas inflammatory or dysfunctional phenotypes may promote vascular and neural injury. BBB integrity further modifies this balance by regulating monocyte–endothelial interactions, perivascular accumulation, and access to AD-associated brain lesions. Thus, disease stage, monocyte phenotype, and BBB status together may determine whether peripheral myeloid responses contribute predominantly to clearance or inflammatory amplification.

### 7.4 Adaptive immune dysregulation and chronic neuroinflammation

Adaptive immune changes add another layer to the BBB–peripheral immune loop. AD develops over decades, and chronic exposure to Aβ, tau-related injury, vascular inflammation, and systemic immune aging may reshape T-cell and B-cell compartments. These changes may sustain low-grade inflammatory states and alter communication between the periphery and CNS. T-cell exhaustion has been associated with cognitive status and amyloid accumulation in AD (30). This suggests that chronic immune stimulation may accompany AD progression. Exhausted T cells may show altered cytokine production, impaired proliferative capacity, and increased expression of inhibitory receptors. Although exhaustion is often interpreted as reduced immune function, it can also indicate persistent antigenic or inflammatory stimulation. In AD, such stimulation may arise from amyloid-related signals, neurodegeneration-associated antigens, systemic inflammation, or chronic vascular injury.

Broader adaptive immune changes are also associated with clinical progression of AD (31). These changes may involve shifts in T-cell subsets, B-cell populations, immune aging signatures, activation states, and cytokine networks. Adaptive immune remodeling may not only reflect disease severity but may also influence progression by regulating monocyte function, endothelial activation, and inflammatory tone. BBB dysfunction may determine how adaptive immune changes affect the CNS. When the BBB is intact, peripheral T-cell and B-cell alterations may influence the brain mainly through soluble mediators or indirect systemic effects. When the BBB is compromised, adaptive immune signals may more readily affect CNS border regions, perivascular spaces, cerebrospinal fluid compartments, or even selected parenchymal niches. In this context, T-cell-derived cytokines may influence endothelial cells, microglia, astrocytes, and monocyte recruitment.

BBB biomarkers appear to modulate the relationship between peripheral immune markers and AD outcomes (17). This supports a model in which adaptive immune dysregulation becomes more clinically relevant when barrier integrity is impaired. For example, patients with both T-cell exhaustion and BBB leakage may be more vulnerable to chronic neuroinflammatory amplification than patients with immune changes but preserved barrier function. Adaptive immune dysregulation may also contribute to immune aging in AD. Older individuals often exhibit expansion of memory and senescent T-cell populations, reduced naive T-cell reserves, impaired immune flexibility, and chronic inflammatory signaling. AD may accelerate or reshape these age-related immune changes. In turn, immune aging may reduce the ability to resolve inflammation, impair tissue repair, and promote persistent vascular and glial activation.

Therefore, adaptive immune changes should be integrated into the inflammation-centered model of AD. They may sustain chronic inflammatory states, regulate innate immune-cell behavior, influence BBB activation, and contribute to disease heterogeneity. This does not imply that all adaptive immune responses are harmful. Some T-cell or B-cell functions may be protective or regulatory. The key issue is whether adaptive immunity remains balanced or shifts toward chronic dysregulation, exhaustion, senescence, and inflammatory amplification.

### 7.5 Downstream consequences: synaptic dysfunction, neurodegeneration, and cognitive decline

The downstream consequences of BBB dysfunction and peripheral immune activation converge on glial activation, synaptic injury, neuronal dysfunction, brain atrophy, and cognitive decline. Inflammation in AD should not be viewed merely as a secondary response to protein aggregation. Instead, chronic neurovascular and immune inflammation can actively drive disease progression by damaging the cellular systems required for neuronal survival and synaptic function. BBB dysfunction can promote synaptic injury through several mechanisms (183–186). Entry of plasma-derived proteins and inflammatory mediators can activate microglia and astrocytes. Activated microglia can release cytokines, reactive oxygen species, and complement-related factors that contribute to synaptic pruning and neuronal stress. Reactive astrocytes can lose homeostatic functions, including metabolic support, neurotransmitter regulation, and vascular coupling. Endothelial dysfunction can impair cerebral perfusion and reduce the ability of blood vessels to match metabolic supply with neuronal activity. Together, these processes create an inflammatory and metabolically unstable environment that compromises synaptic function.

Clinical evidence supports the relevance of BBB dysfunction to cognitive impairment and AD (15). BBB permeability is also associated with neuroinflammatory profiles in AD, indicating that barrier dysfunction may help explain why some patients show stronger inflammatory signatures than others (16). These observations support the idea that BBB disruption is clinically meaningful and may contribute to cognitive decline through inflammatory and vascular mechanisms. Peripheral immune remodeling may further contribute to neurodegeneration. T-cell exhaustion is associated with cognitive status and amyloid accumulation, while broader adaptive immune changes are linked with clinical AD progression (30, 31). These findings suggest that systemic immune status may reflect or influence neurodegenerative burden. When combined with BBB dysfunction, peripheral immune changes may have greater potential to affect CNS inflammation and neuronal vulnerability.

BBB-related biomarkers also modulate the associations between peripheral immunity and AD pathology, brain atrophy, and cognitive function (17). This provides direct support for the integrated model: peripheral immune changes and BBB dysfunction are not separate disease features but interacting determinants of brain injury. BBB impairment may determine whether systemic immune signals remain peripheral or become translated into neuroinflammation, atrophy, and cognitive decline. In this model, AD progression is driven by a pathogenic loop. Aβ, tau, aging, and vascular stress initiate neurovascular injury. BBB leakage allows peripheral inflammatory mediators and immune-cell signals to affect the CNS. Glial activation and oxidative stress further damage the BBB and neurons. Peripheral monocytes and adaptive immune cells participate in both protective clearance and chronic inflammatory amplification. The final result is synaptic dysfunction, neuronal injury, brain atrophy, and cognitive deterioration. Recognizing this loop provides a rationale for targeting not only Aβ and tau, but also BBB integrity, vascular inflammation, and systemic immune dysregulation.

## 8 Therapeutic and biomarker implications

### 8.1 Targeting BBB stability

If BBB dysfunction actively contributes to AD progression, then preserving BBB stability may represent a therapeutic strategy. BBB-targeted therapy should not be viewed as a replacement for anti-Aβ or anti-tau approaches. Instead, it may complement proteinopathy-directed interventions by reducing the vascular and inflammatory environment that promotes neurodegeneration. Several BBB-protective mechanisms have emerged from recent studies. Activation of endothelial Wnt/β-catenin signaling can mitigate BBB dysfunction in AD models, suggesting that restoration of endothelial barrier identity may protect the neurovascular unit (67). This pathway is especially attractive because it directly supports tight junction integrity and BBB-specific endothelial programs. However, systemic activation of Wnt signaling may carry risks, so future strategies should aim for endothelial-specific or brain-targeted modulation.

Endothelial DR6 is another potential target. Evidence suggests that DR6 is involved in BBB malfunction in AD and may regulate BBB functional protein expression under Aβ stress (106). If DR6 supports endothelial resilience, then restoring or preserving DR6-related signaling may help maintain barrier function. Additional mechanistic studies are needed to define whether DR6 modulation is protective across disease stages and whether it can be safely targeted *in vivo*. ANGPT2 represents a vascular-destabilizing target. Because ANGPT2 can promote BBB dysfunction and neuroinflammation in AD, inhibition of ANGPT2-related signaling may help stabilize endothelial junctions and reduce vascular inflammation (49). This strategy may be particularly relevant for patients with vascular-inflammatory AD features, such as BBB leakage, endothelial activation, or elevated inflammatory biomarkers. Inhibition of 15-PGDH provides proof-of-concept that preserving BBB function can protect brain health. In experimental models, 15-PGDH inhibition blocked BBB deterioration and protected mice from AD-related and injury-related brain pathology (107). This finding supports the broader principle that BBB deterioration is modifiable and that vascular-barrier protection may have therapeutic value.

Overall, targeting BBB stability may involve several complementary strategies: enhancing protective endothelial signaling, suppressing vascular destabilization, preserving pericyte function, reducing endothelial senescence, limiting inflammatory cytokine-induced junctional disruption, and modulating BBB-associated immune cells. The major translational challenge is to determine when these interventions should be applied. BBB-stabilizing therapy may be most effective early in the AD continuum, before extensive neuronal loss and irreversible synaptic damage occur.

### 8.2 Targeting vascular inflammation and endothelial immune signaling

Vascular inflammation is a major mechanism connecting BBB dysfunction with peripheral immune activation. Brain endothelial cells are not passive structural components; they actively sense cytokines, chemokines, Aβ, oxidative stress, metabolic changes, and immune-cell signals. In response, they can alter junctional proteins, transporter activity, adhesion molecule expression, and inflammatory mediator release. Therefore, endothelial immune signaling represents a druggable interface between systemic inflammation and CNS pathology. Type I interferon signaling is one important example. Increased type I interferon signaling can impair brain endothelial barrier integrity in AD models by reducing junctional proteins and increasing endothelial leakiness (77). Targeting excessive interferon-related endothelial activation may therefore help preserve BBB integrity and reduce inflammatory signal transmission into the CNS. However, interferon pathways also have important roles in host defense, so therapeutic modulation would need to be selective and carefully timed.

ANGPT2-mediated vascular inflammation is another relevant pathway. ANGPT2 destabilizes endothelial function and has been shown to aggravate AD by promoting BBB dysfunction and neuroinflammation (49). Inhibition of ANGPT2 or restoration of ANGPT1/Tie2 vascular-stabilizing balance may reduce permeability and inflammatory endothelial activation. Such approaches may be particularly useful in patients with vascular injury or BBB leakage. Senescence-associated inflammatory programs also represent potential targets. Vascular senescence and leak are early features of BBB breakdown in AD models (44). Senescent endothelial or perivascular cells may release cytokines, chemokines, proteases, and oxidative mediators that damage barrier integrity and activate glia (44, 187). Targeting senescence-related pathways may therefore reduce chronic vascular inflammation and improve neurovascular resilience. Potential approaches include senomorphic strategies that suppress the senescence-associated secretory phenotype, antioxidant or metabolic interventions, and selective targeting of senescent neurovascular cells; however, these approaches remain experimental and require validation in AD-specific models (65, 187). Targeting vascular inflammation may also be valuable because endothelial inflammatory activation can simultaneously influence BBB permeability, leukocyte–endothelial interactions, and downstream glial responses. Type I interferon signaling, for example, directly impairs brain endothelial junctional proteins and increases barrier leakiness in experimental AD models (77), whereas ANGPT2-mediated suppression of TIE2 signaling promotes vascular leakage and neuroinflammation (49). These findings support the broader concept that reducing endothelial inflammatory activation may interrupt multiple components of the BBB–neuroinflammatory cascade.

A key future direction is to identify vascular-inflammatory biomarkers that can guide patient stratification and therapeutic selection. Patients with evidence of BBB leakage, endothelial inflammatory activation, elevated ANGPT2, interferon-related signatures, or vascular senescence may represent candidate populations for vascular-directed interventions (44, 49). Clinical biomarker evidence further indicates that BBB-related markers modify or mediate associations between peripheral immunity and AD pathology, brain atrophy, and cognitive function, supporting the potential value of integrated BBB–immune profiling for precision stratification (17). However, whether these biomarker profiles can predict response to endothelial- or BBB-targeted therapies remains to be established.

### 8.3 Peripheral immune biomarkers for patient stratification

Peripheral immune biomarkers offer an accessible way to capture systemic inflammatory and immune-aging processes in AD. Because blood is easier to obtain than cerebrospinal fluid or brain tissue, peripheral immune profiling may be useful for patient stratification, longitudinal monitoring, and therapeutic response prediction. However, peripheral immune biomarkers should be interpreted in relation to BBB integrity, because BBB status may determine whether systemic immune changes influence the CNS. Peripheral monocyte function is one promising biomarker category. Monocyte-mediated Aβ clearance and transport correlate with AD progression, suggesting that Aβ-handling capacity may reflect disease-relevant immune function (23). Reduced or altered monocyte Aβ clearance could indicate impaired peripheral buffering of Aβ, defective phagocytosis, or immune dysfunction. Conversely, excessive monocyte activation may suggest inflammatory risk.

Plasma Aβ aggregate-induced monocyte activation in MCI provides another potential early biomarker axis (24). If circulating Aβ aggregates activate monocytes before dementia develops, then measuring both Aβ aggregate burden and monocyte activation state may help identify individuals at higher risk of progression. Such markers may complement classical amyloid PET, CSF Aβ, phosphorylated tau, and neurodegeneration biomarkers by capturing a peripheral inflammatory response to AD-related pathology. Adaptive immune biomarkers are also relevant. T-cell exhaustion markers are associated with cognitive status and amyloid accumulation (30). Broader adaptive immune changes are associated with clinical progression of AD (31). These immune signatures may reflect chronic antigenic stimulation, immune aging, systemic inflammation, or disease-related immune remodeling. They may help define patients with immune-dysregulated AD phenotypes. BBB-related biomarkers are essential for interpreting peripheral immune markers. Evidence that BBB biomarkers modulate associations between peripheral immunity and AD outcomes suggests that immune biomarkers may be most informative when combined with barrier markers (17). For example, elevated monocyte activation may have different implications in a patient with intact BBB compared with a patient with marked BBB leakage. In the latter, peripheral inflammation may more readily amplify CNS inflammation and neurodegeneration.

Therefore, patient stratification should incorporate multiple immune-barrier dimensions: monocyte Aβ-handling capacity, monocyte activation state, T-cell exhaustion markers, adaptive immune signatures, endothelial injury markers, BBB permeability markers, and neuroinflammatory biomarkers. Such integrated profiling may identify vascular-inflammatory AD subtypes and help guide anti-inflammatory, vascular-protective, or immune-modulatory interventions.

### 8.4 Integrating BBB and immune biomarkers with established AD biomarker frameworks

The ATN framework, based on amyloid pathology, tau pathology, and neurodegeneration, has greatly improved the biological classification of AD. However, ATN does not fully capture vascular dysfunction, BBB impairment, neuroinflammation, or peripheral immune remodeling. Because these processes may strongly influence disease progression and treatment response, BBB and immune biomarkers should be integrated with classical ATN markers. Clinical evidence links BBB dysfunction with cognitive impairment and AD, supporting the inclusion of barrier-related measures in AD assessment (15). BBB permeability is also associated with distinct neuroinflammatory profiles, indicating that barrier markers may help define inflammatory heterogeneity within AD (16). Moreover, BBB biomarkers modulate associations between peripheral immunity and AD pathology, brain atrophy, and cognition, suggesting that barrier status can influence how systemic immune changes are translated into neurodegenerative outcomes (17).

An expanded biomarker framework could therefore include amyloid, tau, neurodegeneration, BBB dysfunction, and immune activation. For example, an ATN-positive patient with low BBB leakage and minimal immune activation may have a predominantly proteinopathy-driven phenotype. In contrast, a patient with biomarker-supported AD who also shows prominent BBB permeability, endothelial dysfunction, peripheral monocyte activation, and adaptive immune dysregulation may represent a candidate vascular-inflammatory phenotype. Importantly, the proposed phenotype is defined by the convergence of vascular-barrier and immune abnormalities rather than by any single biomarker. These subtypes may differ in progression rate, cognitive trajectory, comorbid vascular risk, and therapeutic responsiveness. This integrated approach may also improve clinical trial design. Anti-amyloid therapies may be less effective in patients whose cognitive decline is strongly driven by vascular inflammation or peripheral immune activation. Conversely, BBB-stabilizing or immune-modulatory therapies may be most effective in patients with biomarker evidence of barrier dysfunction and immune activation. Combining BBB and immune biomarkers with ATN markers could therefore help enrich clinical trials for biologically appropriate participants. In clinical practice, such integration may eventually support more personalized AD management. Patients with strong vascular-inflammatory signatures may benefit from aggressive control of vascular risk factors, anti-inflammatory strategies, endothelial-protective interventions, or immune-modulatory approaches. Patients with predominant amyloid/tau pathology but limited vascular inflammation may be better suited for proteinopathy-targeted therapies. Importantly, these categories are not mutually exclusive; many patients may have mixed pathology and require combination strategies.

Overall, incorporating BBB and immune biomarkers into AD classification could shift the field from a proteinopathy-centered model toward a multidimensional disease framework. This would better reflect the biological complexity of AD and may identify treatable vascular-inflammatory mechanisms that are not captured by amyloid and tau biomarkers alone. The BBB–peripheral immune axis provides several potential biomarkers and therapeutic targets that may complement classical amyloid, tau, and neurodegeneration markers. Emerging candidates related to BBB stability, endothelial inflammation, peripheral immune activation, and vascular-inflammatory AD subtyping are summarized in **Table 3**.

**Table 3:** Potential biomarkers and therapeutic targets related to the BBB–peripheral immune axis in AD.

Category	Candidate biomarker/target	References	Biological implication	Potential application
BBB integrity	BBB permeability or BBB-related biomarkers	(15–17)	Reflects barrier leakage, vascular injury, and inflammatory vulnerability.	Patient stratification; monitoring vascular-inflammatory AD subtype.
Pericyte injury	CD36/PINK1/Parkin-mediated mitophagy-dependent ferroptosis	(55, 108)	Links Aβ toxicity to pericyte loss, mitochondrial dysfunction, ferroptosis, and BBB breakdown.	Targeting pericyte protection and ferroptosis may stabilize the BBB.
Endothelial protection	Wnt/β-catenin signaling	(67)	Maintains endothelial barrier phenotype and tight junction integrity.	BBB-protective therapeutic strategy; possible combination with anti-Aβ therapy.
Endothelial inflammation	Type I interferon signaling	(77)	Promotes endothelial leakiness and junctional protein loss.	Targeting excessive endothelial inflammatory signaling.
Vascular aging	Vascular senescence markers	(44)	Indicates age-related BBB vulnerability and inflammaging-associated leak.	Early detection of vascular-inflammatory risk; senescence-modulating interventions.
Neurovascular remodeling	Brain/BBB proteomic signatures	(109)	Captures coordinated molecular remodeling of BBB and brain compartments.	Discovery of biomarker panels and vascular therapeutic targets.
Vascular-cell states	Endothelial and angiogenesis-related transcriptomic signatures	(34)	Reveals impaired angiogenesis and BBB-related endothelial alterations.	Cell-type-specific biomarker discovery and mechanistic classification.
Gliovascular communication	Endothelial–pericyte–astrocyte interaction signatures	(35)	Reflects disrupted multicellular support of BBB function.	Identification of neurovascular-unit therapeutic nodes.
Endothelial regulator	DR6	(106)	Regulates BBB functional proteins and endothelial responses to Aβ stress.	Potential BBB-stabilizing molecular target.
Vascular inflammatory mediator	ANGPT2	(49)	Promotes BBB dysfunction and neuroinflammation.	Candidate target for vascular-inflammatory AD intervention.
Barrier-protective intervention	15-PGDH inhibition	(107)	Blocks BBB deterioration and protects brain function in experimental models.	Proof-of-concept for BBB-centered therapy.
Blood–CSF interface	Choroid plexus transcriptional abnormalities	(130)	Suggests early blood–CSF barrier and immune-interface disruption.	Expands biomarker discovery beyond the classical BBB.
Peripheral innate immunity	Monocyte Aβ clearance and transport capacity	(23)	Reflects functional interaction between peripheral monocytes and Aβ pathology.	Blood-based immune biomarker; patient stratification.
Prodromal immune activation	Plasma Aβ aggregate-induced monocyte activation	(24)	Indicates early peripheral innate immune activation in MCI.	Early-risk biomarker and monitoring of immune activation.
Peripheral myeloid recruitment	Monocyte-derived cells in plaques or parenchyma	(158, 160)	May represent both Aβ-clearing capacity and barrier/perivascular immune remodeling.	Distinguishing protective versus harmful myeloid responses.
Adaptive immunity	T-cell exhaustion markers	(30)	Associated with amyloid accumulation and cognitive status.	Immune-aging biomarker; monitoring chronic immune dysfunction.
Immune progression signature	Adaptive immune-cell changes	(31)	Associated with clinical progression of AD.	Stratifying patients by immune remodeling status.
Integrated vascular-immune subtype	Combined BBB and peripheral immune markers	(16, 17)	Defines patients in whom BBB leakage and peripheral immune activation jointly shape AD pathology.	Development of vascular-inflammatory AD subtypes and combination therapies.

## 9 Challenges and future directions

### 9.1 Defining causality and temporal order

Although accumulating evidence supports a close association among BBB dysfunction, peripheral immune activation, neuroinflammation, and cognitive decline in AD, the causal and temporal relationships among these processes remain incompletely defined. A central unresolved question is whether BBB dysfunction precedes amyloid and tau pathology, accelerates their progression, or develops as a parallel process driven by aging, vascular risk, and systemic inflammation. It is also possible that the relative timing differs among patients, depending on genetic background, vascular comorbidities, immune status, metabolic disease, and disease stage. Clinical evidence has linked BBB impairment with cognitive dysfunction and AD-related pathology, suggesting that BBB damage is biologically and clinically meaningful (15). However, cross-sectional associations cannot establish whether barrier dysfunction is a cause or consequence of neurodegeneration. BBB breakdown may occur downstream of Aβ deposition, tau-related neuronal injury, and glial activation. Conversely, BBB leakage may also precede overt dementia by impairing Aβ clearance, exposing the CNS to plasma-derived inflammatory mediators, and promoting glial activation. These possibilities are not mutually exclusive.

Aging-related vascular senescence further complicates the temporal model. Vascular senescence and leak have been identified as early features of BBB breakdown in AD models, suggesting that cerebrovascular aging may prime the BBB before extensive neurodegeneration is established (44). This supports a model in which aging and vascular stress create a vulnerable neurovascular environment, while Aβ, tau, systemic inflammation, and metabolic dysfunction further accelerate barrier injury. In this scenario, BBB dysfunction may act as both an early permissive factor and a later amplifier of AD pathology. BBB permeability is also associated with different neuroinflammatory profiles in AD, indicating that barrier dysfunction may define inflammatory heterogeneity among patients (16). This raises another important question: does BBB dysfunction drive specific inflammatory phenotypes, or do distinct inflammatory phenotypes damage the BBB? A bidirectional relationship is likely. Peripheral and CNS inflammation can impair endothelial junctions and vascular integrity, while BBB leakage can enhance exposure of brain-resident cells to inflammatory mediators.

Recent evidence that BBB biomarkers modulate the associations between peripheral immune biomarkers and AD pathology, brain atrophy, and cognitive function further supports the concept that BBB status determines how systemic immunity influences the brain (17). However, longitudinal data are needed to clarify whether changes in BBB biomarkers occur before, during, or after peripheral immune activation and neurodegeneration. Future studies should therefore prioritize longitudinal designs that integrate BBB imaging, blood and CSF biomarkers, amyloid and tau markers, immune profiling, neuroimaging, and cognitive assessment. Ideally, such studies should begin in cognitively normal older adults or individuals with subjective cognitive decline and follow them through MCI and dementia stages. Repeated measurements will be essential to define whether BBB leakage predicts subsequent peripheral immune activation, glial inflammation, amyloid/tau progression, brain atrophy, or cognitive decline. Without this temporal information, it will remain difficult to determine whether BBB dysfunction is primarily a biomarker, mediator, amplifier, or therapeutic target in AD.

### 9.2 Distinguishing protective versus harmful peripheral immune responses

Another major challenge is distinguishing protective peripheral immune responses from harmful inflammatory processes. Peripheral immunity is not uniformly detrimental in AD. Monocytes, macrophages, T cells, and other immune-cell populations may perform beneficial functions under certain conditions, including Aβ clearance, debris removal, immune surveillance, vascular repair, and resolution of inflammation. However, these same cells may also promote pathology when they become chronically activated, senescent, exhausted, or excessively inflammatory. Peripheral monocytes provide a clear example of this duality. Their ability to clear and transport Aβ correlates with AD progression, suggesting that monocyte Aβ-handling capacity may have protective or disease-modifying relevance (23). If monocytes efficiently bind, internalize, degrade, or transport Aβ, they may reduce toxic Aβ burden and support systemic clearance. Conversely, impaired monocyte phagocytosis or dysfunctional lysosomal processing may weaken this protective mechanism.

At the same time, Aβ aggregates in plasma can activate peripheral monocytes in MCI, indicating that circulating Aβ species may also drive inflammatory monocyte responses early in the disease process (24). Activated monocytes may release cytokines, chemokines, reactive oxygen species, and endothelial-activating mediators. These factors can contribute to systemic inflammation, vascular dysfunction, and BBB impairment. Thus, Aβ–monocyte interactions may be protective when they promote clearance but harmful when they induce chronic inflammatory activation. Animal studies further highlight this complexity. Peripheral monocyte-derived cells can enter the AD brain and counter amyloid plaque pathogenesis in mouse models, suggesting a potentially beneficial plaque-clearing role (158). However, immune-cell recruitment to the brain also implies altered BBB or perivascular immune regulation. If recruitment becomes excessive or poorly controlled, infiltrating cells may amplify neuroinflammation, produce toxic mediators, and damage the neurovascular unit.

Human evidence showing monocyte-derived cells in the hippocampal parenchyma and amyloid plaques of AD cases provides translational support for peripheral myeloid involvement (160). Yet the functional meaning of these cells remains uncertain. They may represent reparative phagocytes, inflammatory macrophage-like cells, antigen-presenting cells, or mixed transitional states. Their impact may differ across brain regions, disease stages, plaque environments, and patient immune backgrounds. Future studies need to move beyond simple classification of peripheral immune responses as “good” or “bad.” Instead, they should define immune-cell states, timing, spatial localization, and functional outputs. Single-cell transcriptomics, spatial proteomics, lineage tracing, fate mapping, and functional assays will be required to distinguish Aβ-clearing monocytes from inflammatory monocytes, reparative macrophage-like cells from tissue-damaging infiltrates, and protective adaptive immune responses from chronic immune exhaustion. This distinction is crucial for therapy. Broad immunosuppression may eliminate beneficial immune functions, whereas precise immune modulation could enhance Aβ clearance and tissue repair while limiting inflammatory injury. Future studies should therefore determine monocyte function in a stage-resolved and phenotype-specific manner while simultaneously assessing BBB integrity, because these three variables are likely to jointly determine whether peripheral myeloid responses are beneficial, neutral, or detrimental.

### 9.3 Improving model systems

Current model systems have provided important mechanistic insight into BBB dysfunction and peripheral immune activation in AD, but they also have major limitations. Many AD mouse models overexpress familial AD-associated mutations or develop amyloid pathology without fully reproducing the complexity of sporadic late-onset AD. They often incompletely model human vascular aging, immune aging, tau pathology, metabolic comorbidities, sex differences, and chronic systemic inflammation. Because most human AD cases occur in aged individuals with mixed vascular, metabolic, inflammatory, and proteinopathic pathology, model limitations may affect the translational relevance of BBB and immune findings.

Proteomic studies in AD mouse models have shown coordinated alterations in the brain and BBB compartments, supporting the value of animal models for mapping disease-associated molecular changes (109). However, mouse BBB biology, immune-cell recruitment patterns, and vascular aging may differ from those in humans. Therefore, findings from animal models should be validated in human tissue and human-derived experimental systems whenever possible. Single-nucleus transcriptomic studies have revealed endothelial and vascular-cell alterations related to impaired angiogenesis and BBB function in AD (34). These approaches are valuable because they identify cell-type-specific disease states that are difficult to detect in bulk tissue analysis. Similarly, gliovascular transcriptomic studies have shown disrupted communication among endothelial cells, pericytes, astrocytes, and other neurovascular components (35). Such multi-cellular analyses should be further combined with spatial technologies to preserve anatomical context, especially around vessels, plaques, perivascular spaces, and inflammatory niches.

The choroid plexus and blood–CSF barrier also deserve greater attention. Early transcriptional and cellular abnormalities in the choroid plexus of AD models suggest that CNS barrier dysfunction extends beyond the classical BBB (130). Future models should therefore include multiple CNS barrier systems, including the BBB, blood–CSF barrier, meningeal barriers, perivascular drainage pathways, and glymphatic clearance routes. Human brain tissue remains essential for validation. Evidence that monocyte-derived cells invade the hippocampal parenchyma and amyloid plaques in human AD tissue highlights the importance of human neuropathological studies (160). However, postmortem tissue provides only a static endpoint and may be influenced by agonal factors, disease duration, and comorbidities. Combining postmortem tissue with longitudinal clinical cohorts, imaging, blood biomarkers, CSF biomarkers, and induced pluripotent stem cell-derived models may provide a more complete picture.

Future experimental platforms should include human BBB-on-chip systems, brain endothelial–pericyte–astrocyte co-cultures, immune-cell transmigration models, vascularized brain organoids, and patient-derived induced pluripotent stem cell systems. These models can help test causal mechanisms under controlled conditions, such as how Aβ, inflammatory cytokines, senescent endothelial cells, monocytes, or T cells alter BBB integrity. They can also be used for drug screening and personalized evaluation of BBB-protective or immune-modulatory interventions.

Ultimately, progress will require integration across model systems. Animal models remain useful for *in vivo* physiology and longitudinal mechanistic studies. Human tissue provides disease relevance. Organoids and BBB-on-chip systems allow mechanistic manipulation. Single-cell and spatial omics define cellular states and tissue organization. Longitudinal clinical cohorts establish temporal relationships and biomarker value. Combining these approaches will be essential to clarify how BBB dysfunction and peripheral immunity shape AD progression.

### 9.4 Defining a proposed vascular-inflammatory AD phenotype

AD is biologically heterogeneous. Although amyloid and tau define the core pathological framework, they do not fully explain differences in clinical progression, inflammatory burden, vascular involvement, treatment response, or cognitive trajectory. An important future direction is to define vascular-immune subtypes of AD based on BBB dysfunction, endothelial inflammation, monocyte activation, adaptive immune remodeling, and glial reactivity. BBB permeability is associated with distinct neuroinflammatory profiles in AD, suggesting that barrier status may identify patients with stronger inflammatory involvement (16). Adaptive immune changes are associated with clinical progression of AD, indicating that systemic immune remodeling may track disease stage or severity (31). T-cell exhaustion is associated with cognitive status and amyloid accumulation, further supporting the link between adaptive immune dysfunction and AD-related outcomes (30). In addition, BBB biomarkers modulate associations between peripheral immunity and AD pathology, brain atrophy, and cognition, providing a direct rationale for integrating BBB and immune markers into disease subtyping (17).

In this review, we use the term “vascular-inflammatory AD phenotype” to describe a proposed, rather than clinically established, form of biological heterogeneity in which AD-related proteinopathy coexists with prominent BBB/neurovascular dysfunction and peripheral immune dysregulation. Its defining feature is not the presence of vascular or inflammatory abnormalities alone, but their convergence: impaired BBB integrity or endothelial dysfunction occurs together with systemic immune activation and may enhance communication between peripheral inflammatory signals and the CNS. This phenotype should therefore be regarded as a testable stratification framework rather than a formally validated diagnostic subtype. Conceptually, this phenotype may be characterized by three interconnected domains. The first is BBB and neurovascular dysfunction, reflected by increased BBB permeability, endothelial injury or activation, pericyte dysfunction, vascular senescence, and impaired neurovascular homeostasis. The second is peripheral immune dysregulation, including altered monocyte Aβ-handling capacity, excessive monocyte activation, T-cell exhaustion, and broader adaptive immune remodeling. The third is enhanced neuroinflammatory coupling, in which BBB dysfunction permits or amplifies communication between systemic inflammatory signals and CNS-resident microglia and astrocytes. These domains are expected to overlap rather than occur as independent abnormalities. Potential identification of this phenotype would therefore require a multimodal biomarker strategy rather than a single marker. A BBB/vascular module could include imaging-based BBB permeability measures, BBB-related or endothelial injury biomarkers, and markers of vascular inflammatory activation. A peripheral immune module could include monocyte Aβ-clearance and transport capacity, plasma Aβ aggregate-induced monocyte activation, T-cell exhaustion markers, and broader adaptive immune signatures. These measures should be interpreted together with established AD biomarkers to distinguish vascular-inflammatory modifiers from the underlying AD biological process. At present, no validated biomarker threshold or diagnostic algorithm exists for this proposed phenotype; prospective longitudinal studies will be required to determine the most informative marker combinations and clinically meaningful cutoffs.

A vascular-inflammatory AD subtype may be characterized by increased BBB permeability, endothelial activation, vascular senescence, elevated inflammatory cytokines or chemokines, altered monocyte Aβ-handling capacity, monocyte activation, T-cell exhaustion, adaptive immune remodeling, and enhanced glial reactivity. Patients exhibiting this phenotype would be expected to show established AD pathology together with disproportionate vascular-barrier and immune abnormalities. Whether such individuals experience faster cognitive decline, greater vulnerability to systemic inflammatory events, or differential responses to anti-amyloid therapies remains an important hypothesis that requires prospective validation. This subtype concept has therapeutic implications. Patients with strong vascular-inflammatory features may require combination strategies that include BBB stabilization, vascular risk control, endothelial-protective treatment, immune modulation, and classical anti-Aβ or anti-tau approaches. In contrast, patients with predominantly proteinopathy-driven pathology and limited BBB or immune involvement may respond differently. Therefore, vascular-immune subtyping could improve patient selection in clinical trials and help explain variability in therapeutic outcomes.

To develop such subtypes, future studies should integrate multiple biomarker domains. These may include amyloid and tau markers, neurodegeneration markers, BBB permeability imaging, endothelial injury markers, CSF/blood inflammatory markers, monocyte functional assays, T-cell exhaustion markers, adaptive immune profiling, vascular imaging, and cognitive trajectories. Machine-learning approaches may help identify reproducible vascular-immune phenotypes, but these models must be validated across independent cohorts and populations. Importantly, vascular-immune subtyping should not replace existing AD biomarker frameworks. Rather, it should extend them. Amyloid, tau, and neurodegeneration remain essential for defining AD biology, but BBB and immune biomarkers may explain disease modifiers, progression patterns, and treatment responsiveness. This multidimensional framework may better capture the complexity of late-onset AD and support more individualized therapeutic strategies.

## 10 Conclusion

BBB dysfunction and peripheral immune activation represent an interconnected inflammatory axis in AD. Current evidence suggests that Aβ deposition, tau-related stress, aging, vascular senescence, endothelial inflammatory signaling, pericyte injury, and gliovascular remodeling weaken BBB integrity. Once the barrier is compromised, peripheral immune mediators, plasma-derived factors, monocytes, T cells, and other immune signals can communicate more effectively with the CNS. This communication can amplify microglial activation, astrocyte reactivity, oxidative stress, synaptic dysfunction, neuronal injury, brain atrophy, and cognitive decline. The BBB should therefore be viewed not only as a structural barrier but also as an active immunovascular interface. It regulates the extent to which peripheral inflammation affects the brain and may determine whether systemic immune changes remain peripheral or become translated into neuroinflammatory damage. Peripheral monocytes may contribute to Aβ clearance and plaque-associated responses, but they may also promote inflammation when chronically activated or recruited into a dysfunctional vascular environment. Adaptive immune alterations, including T-cell exhaustion and broader immune remodeling, further suggest that AD progression is accompanied by systemic immune dysregulation. This inflammation-centered model expands classical amyloid- and tau-centered views of AD. It does not reject the importance of Aβ or tau, but instead places them within a broader pathogenic network involving vascular injury, BBB leakage, peripheral immune activation, and glial inflammation. Such a framework may help explain why patients with similar amyloid or tau burden can show different clinical trajectories and inflammatory profiles. Targeting the BBB–peripheral immune axis may provide new opportunities for biomarker development, patient stratification, and therapeutic intervention. BBB biomarkers and peripheral immune signatures could be integrated with classical amyloid, tau, and neurodegeneration markers to identify vascular-inflammatory AD subtypes. These subtypes may benefit from combination strategies that stabilize the BBB, reduce endothelial inflammation, preserve beneficial immune clearance, limit harmful chronic immune activation, and complement anti-Aβ or anti-tau therapies. Future research should clarify causality, define temporal relationships, distinguish protective from harmful immune responses, improve human-relevant model systems, and validate vascular-immune biomarkers in longitudinal clinical cohorts. Understanding the BBB–peripheral immune axis may ultimately shift AD research toward a more integrated view of neurodegeneration, in which the brain, vasculature, and immune system are treated as interconnected components of disease progression.
